# Antitrust analysis with upward pricing pressure and cost efficiencies

**DOI:** 10.1371/journal.pone.0227418

**Published:** 2020-01-08

**Authors:** Jéssica Dutra, Tarun Sabarwal

**Affiliations:** 1 Economists Incorporated, Washington, DC, United States of America; 2 Economics Department, The University of Kansas, Lawrence, KS, United States of America; Universitat Jaume I, SPAIN

## Abstract

We investigate the accuracy of UPP as a tool in antitrust analysis when there are cost efficiencies from a horizontal merger. We include merger-specific cost efficiencies in a tractable manner in the model and extend the standard UPP formulation to account for these efficiencies. The efficacy of the new UPP formulations is analyzed using Monte Carlo simulation of 40,000 mergers (8 scenarios, 5,000 mergers in each scenario). We find that the new UPP formulations yield substantial gains in prediction of post-merger prices, and there are substantial gains in merger screening accuracy as well. Moreover, the new UPP formulations outperform the standard UPP formulation at higher thresholds for all the standard cases in the paper. The results are robust to several additional analyses. The results show that including cost efficiencies in a manner guided by the theoretical model may yield substantial improvements in accuracy of UPP as a tool in antitrust analysis.

## Introduction

A central tenet in antitrust policy is that antitrust agencies want to block mergers that are anticompetitive without interfering with ones that are procompetitive. Antitrust agencies spend considerable time, effort, and resources to determine the impact a merger may have on the post-merger competitive landscape.

Standard approaches focus on well-developed tools such as the Herfindahl Hirschman Index (HHI) and full-merger simulations. More recently, Upward Pricing Pressure (UPP), proposed by [1], is being used as a pre-merger screening tool to estimate anticompetitive effects in horizontal mergers. UPP is now included in the U.S. Department of Justice and the Federal Trade Commission Horizontal Merger Guidelines (2010) and used increasingly worldwide—The United Kingdom (2010) incorporates UPP to their horizontal merger assessment guidelines, §§5.4.6–5.4.11, highlighting the need to associate its analysis with price sensitivity of consumers through own and cross-price elasticities; In France (2013), as expressed in *Les lignes directrices relatives au contrôle des concentrations* V.D.2.c.(405-420), not only is UPP adopted, it highlights the need for proper efficiency estimates jointly with it; Brazil (2016) in *Guia Análise de Atos de Concentração Horizontal* §2.5.2. shows that the likelihood of harm from mergers with heterogeneous goods arises from the proximity of substitution (diversion).

UPP measures the first impulse for a merged firm to raise prices. It is derived by comparing the first-order condition of the merged firm with that of the pre-merger firm. The standard formulation is as follows. Suppose firms *i* and *j* merge. Upward pricing pressure (for the good produced by firm *i*) is given by *UPP*_*i*_ = *D*_*ij*_(*P*_*j*_ − *MC*_*j*_). Here *D*_*ij*_ is the diversion matrix, which measures proportion of sales lost by firm *i* that are recaptured by firm *j*, and (*P*_*j*_ − *MC*_*j*_) is the margin for firm *j*. Both are computed at pre-merger values.

The UPP computation has several benefits. It uses information about merging firms only, not other firms in the industry, and therefore, market shares of other firms are not needed. This simplifies its computation. Moreover, it is relatively quick and easy to implement, requires less information than some other measures, and is theoretically grounded.

The UPP computation provides a good measure of the first impulse to raise prices from a merger. Notably, it does not predict post-merger prices or provide an estimate of accuracy of price prediction. Moreover, its standard application does not include cost efficiencies from a merger. When the UPP calculation is high, merging parties argue with antitrust agencies that the UPP calculation should be lowered, because there are cost efficiencies from the merger, but antitrust agencies argue for more realistic numbers and require additional justification. Current practice is to arrive at some reduction to the UPP calculation to account for cost efficiencies. The relation of the magnitude of this reduction to more fundamental principles is typically left unexplored.

Using the theoretical framework in [2], we include cost efficiencies in a tractable manner and derive the related UPP formulations.

The efficacy of the new UPP formulations is analyzed using Monte Carlo simulation for a variety of scenarios: Four demand systems (Logit, Linear, Loglinear, and Almost ideal), two merger-specific, cost complementarity systems (Generalized Leontief and Quadratic), and a total of 40,000 mergers (8 scenarios, 5,000 mergers in each scenario). For each merger, we compute several measures of UPP (including standard and new), compute post-merger equilibrium, and compute effectiveness of UPP in terms of price-prediction accuracy and merger screening accuracy.

We find that with inclusion of merger-specific cost efficiencies and using a more accurate first order approximation to compute UPP, there are substantial gains in prediction of post-merger equilibrium prices. In seven of eight scenarios, UPP based price predictions are within 3 percent of post-merger equilibrium prices at the median, both in absolute and relative terms. (In five scenarios, these are within 0.25 percent.)

Similarly, we find that with the new UPP measures, there are substantial gains in merger screening accuracy. In six of eight scenarios, UPP based merger screens (at 5% price increase threshold) reduce total probability of false positives (flagging a merger for scrutiny incorrectly) and false negatives (letting a merger go through incorrectly) to less than 0.02. (In four scenarios, this is 0.007 or less.)

Finally, to provide a stricter comparison to existing practice, we compare the standard UPP formulation with higher thresholds (of 5%, 10%, and 15% price increase) to the new UPP measures with a 0% threshold. In every scenario, total probability of false positives and negatives is lower for the new UPP measures, and in most cases, there is a large reduction in the total error. The results are robust to several additional analyses, including using F1 score and allowing for more flexible merger-specific efficiencies.

Our results support the continuing use of UPP as a tool in antitrust analysis. Effectiveness and accuracy of UPP increase greatly when merger-specific efficiencies are included in a manner guided by the theoretical model. Indeed, UPP calculations may sometimes be a good substitute for full merger simulations. As UPP provides a conceptual framework that is sometimes easier to explain to a broader audience of antitrust practitioners and legal professionals, and it may be less expensive to implement, this would increase its usefulness to practitioners as well.

The paper proceeds as follows. The next section includes a literature review. Section 3 details the theoretical framework and formulates different measures of UPP used in the analysis. Section 4 describes the Monte Carlo simulations and data generating process. Section 5 presents the results and analysis. Section 6 concludes.

## Literature review

“Antitrust is generally viewed as a public policy aimed at fostering a public good: that is, competition” [3, p. xviii]. Competition agencies ought to provide effective and timely evaluations of potential pro- and anti-competitive effects of proposed mergers. In the United States, Section 7 of the Clayton Act is the principal federal substantive law governing mergers, acquisitions, and joint ventures, and which deems unlawful the acquisition of a firm or its stocks such that “the effect of such acquisition may be substantially to lessen competition, or to tend to create a monopoly.” Despite the clarity of the established goal, in reality things may not be as straightforward. Determining *ex ante* whether a merger will generate anticompetitive effects if allowed to go through requires a fair amount of analysis—theoretical, empirical, and institutional.

Whenever screening potential mergers, the antitrust agencies attempt to disentangle and evaluate two potential sources of anticompetitive effects: unilateral and coordinated [4, 5]. Unilateral effects arise whenever a newly merged firm engages in decision-making strategies characteristic of higher market power (in terms of ability to restrict output and price above the competitive level) due to consolidation. Coordinated effects, on the other hand, focus on changes in likelihood of collusion, either express or tacit, within a market due to consolidation—This change may manifest itself by the elimination of a maverick (*i.e*. a firm that seems to be a natural disruptor of tacit collusion, by constantly setting lower prices for example) or by simply reducing the number of players and facilitating that a collusive equilibrium might occur. This paper focuses on unilateral effects, specifically those measuring incentive of consolidated firm to raise prices post-merger.

Since the development of the first merger guidelines in the United States in 1968 until its latest version in 2010, the focus has been to identify and guide the processes by which potential anticompetitive effects may arise. [6] reviews the main points of evolution and change over time, and highlights what has been learned in these four decades with respect to merger analysis. An overall trend is the shift in focus from narrower market concentration concerns to anticompetitive effects evaluated more holistically.

Market concentration methods such as HHI continue to retain a prominent position in antitrust analysis. A well-known limitation of HHI or any other market-share based index for merger screening is the difficulty of market definition for natural competitors of differentiated goods [7–9]. This has fueled demand for alternative measures of anticompetitive effects arising from a merger.

Full merger simulations, an application of formal structural game-theoretical models to determine and predict unilateral anticompetitive effects, are expensive, time consuming, and depend on strong assumptions, prior beliefs, and available data [10–12]. [13] test the accuracy of merger simulation and examine potential sources of differences in the simulated and directly estimated price effect. A substantial source of these differences is estimated to be changes in the cost structure—either a reduction or a slight augmentation in marginal costs. [14] show that a merger simulation represents a major improvement due to its technical potential, nevertheless it should still be combined with alternative instruments of competition policy.

Due to the costly nature of full-merger simulations, it is useful to have alternative merger screening tools that are less expensive, quick, reliable, and theoretically grounded. UPP is being used increasingly in this regard as a pre-merger screening tool for horizontal mergers (*e.g*., E.I. du Pont de Nemours & Co., 353 U.S. at 592, City of NY v. Group Health Incorp., 649 F.3d 151 (2d Cir. 2011), FTC v. Lundbeck, Inc., 650 F.3d 1236 (8th Cir. Aug 19, 2011)).

### UPP and first order approximation

[1] develop upward pricing pressure as an index of likely unilateral effects from a merger, measured in monetary value of price increase resulting from a merger of horizontal competitors with partially differentiated goods. UPP indicates existence and strength of unilateral anticompetitive effects through an incentive to increase price of the goods produced by a merged firm. UPP doesn’t claim to provide the exact amount that the merged firm will raise prices in post-merger equilibrium, but rather provides a measure of the initial incentive to do so, holding fixed other economic environment parameters, such as price and level of output of other firms, demand determinants, and so on. Therefore once the market re-equilibrates to a new post-merger equilibrium, the actual change in prices may be different from a change in first response.

This difference between first impulse to raise prices and post-merger equilibrium prices has been a source of debate in the literature. [15] prefer measures that predict post-merger equilibrium prices accurately, saying “hill’s height is unrelated to how steep the hill is at its base.” [16] point out that the first impulse has important information about final post-merger prices, saying “a ball that is kicked harder might not travel further […] but as a general matter hard-kicked balls tend to go further.”

[17] proposes a first-order approximation approach as an alternative to functional form simulation. [2] generalize the first-order approximation approach and show that it can be used to derive and improve the theoretical formulation of UPP. In particular, including a demand pass-through matrix makes the UPP computation more theoretically accurate as a first-impulse to raise prices. Their approach includes multi-product firms and is independent of particular functional forms for demand or costs. [18] investigates UPP computations in different directions, including how to consider pricing pressures in a merger that may alter the quality of products of merging firms. [19] study unilateral pricing incentives in vertical mergers taking under consideration cost efficiencies both upstream and downstream.

[20] investigate the accuracy of the first-order approximation in a Monte Carlo simulation of merger analysis in oligopoly models and compare it to the corresponding post-merger equilibrium. They find improvements in accuracy when using UPP with the first-order approximation. The employment of pass-through in merger simulation techniques [7, 21, 22] has been much studied in academic settings as well as employed by practitioners in a litigious setting. [23] focus on the role pass-through may play in improving the prediction of post merger prices.

[24] evaluates the performance of UPP as a merger screening tool in contrast to standard structural merger simulation by generating hypothetical mergers using US airline industry data. She documents favorable results in “best case scenario” when full information is available, as well as within correct decile predictions. [25] compares UPP with many other merger screening tools showing that “first-order pricing incentives of merged hospitals (in particular, WTP and UPP) are more accurate at flagging mergers that are potentially anticompetitive than the traditional tools of market definition and concentration measurement.”

[26] compare results from UPP and first order approximation with those obtained from merger simulation for a variety of economic environments as well as different practitioner conditions (such as mis-observed demand elasticity, wrong functional form of demand and pass-through). They show that UPP is accurate with standard log-concave demand systems, slightly understating the effect in demands with greater convexity. Notably, predicted errors with UPP do not exceed in magnitude those from merger simulation with misspecified models or with imprecise demand elasticities. [26] do not include production costs in their setting, normalizing costs to be zero. This rules out consideration of cost efficiencies, which is the main focus of our work.

Jointly, these papers provide a compelling argument for adopting first order approximation techniques in merger analysis. They perform well as compared to full-blown merger simulations, are less computationally heavy, and require less information under a cost variety of different scenarios. This strand of the UPP literature typically does not include efficiencies from a merger.

### Cost efficiencies and UPP

Efficiencies are often used as a motivation for mergers. Indeed, HMG (2010) state that “a primary benefit of mergers to the economy is their potential to generate significant efficiencies and thus enhance the merged firm’s ability and incentive to compete, which may result in lower prices, improved quality, enhanced service, or new products.” Moreover, “[i]n a unilateral effects context, incremental cost reductions may reduce or reverse any increases in the merged firm’s incentive to elevate price” and thus, at least in principle, should be incorporated into post-merger price predictions relating to unilateral effects.

Nevertheless, these guidelines caution that efficiency claims alone are not enough to justify a merger, because “[e]ven when efficiencies generated through a merger enhance a firm’s ability to compete, however, a merger may have other effects that may lessen competition and make the merger anticompetitive” (Horizontal Merger Guidelines (2010) §4). Indeed, antitrust agencies are very skeptical of efficiency claims of pro-competitive effects in rule of reason analysis (For a comprehensive review of the historical evolution of antitrust policy regarding merger efficiency claims in the United States and European Union, see [27, Chapter 3]. [28] explains in a little more detail specificities about the German case and [29] goes through the asymmetries and implicit bias of competition agencies both in the U.S. and European Union with regard to the burden of proof). In order to be considered seriously, efficiency claims by the merging parties have to be merger-specific and verifiable.

“The Agencies credit only those efficiencies likely to be accomplished with the proposed merger and unlikely to be accomplished in the absence of either the proposed merger or another means having comparable anticompetitive effects. These are termed merger-specific efficiencies[…] Efficiency claims will not be considered if they are vague, speculative, or otherwise cannot be verified by reasonable means.[…] Cognizable efficiencies are merger-specific efficiencies that have been verified and do not arise from anticompetitive reductions in output or service. Cognizable efficiencies are assessed net of costs produced by the merger or incurred in achieving those efficiencies.” Department of Justice and Federal Trade Commission Horizontal Merger Guidelines (2010)

This has historically been interpreted to exclude most efficiency claims related to economies of scale, because scale economies can at least hypothetically be obtained through means other than a merger [30].

Indeed, in the standard formulation, the total cost of the merged firm is the sum of cost functions of the merging firms, eliminating cross-firm cost complementarities that typically form the basis of merger-specific efficiencies. As shown by [31], mergers in Bertrand-type markets with differentiated products yield higher prices in the absence of efficiencies.

[1] suggest accommodating efficiencies by including a “standard efficiency-credit”, as in [32], to serve as a proxy for merger-specific efficiencies. As mentioned in [33], a limitation is that the “model would still lack empirical verification,” and therefore, should not be used in lieu of merger-specific efficiencies. UPP computations may be extended to other types of mergers, including vertical mergers and mergers among firms that produce same type components of a composite good, for example, as considered in [34].

We revisit the base model used to derive UPP and include merger-specific cost efficiencies in the model. Using the theoretical framework in [2], we include efficiencies in a tractable manner and derive the related UPP formulations. In our framework, cost efficiencies are made merger-specific by requiring these to be zero if output of either firm in the merger is zero. In other words, cost efficiencies are activated only for the merged firm and only when outputs of both merging firms are positive. The new formulations are naturally connected to existing formulations and show how to modify existing formulations to account for cost efficiencies in a transparent manner. Details are included in the next section.

## Theoretical framework

Following [2], let *I* = {1, …, *N*} be the set of *N* ≥ 2 firms producing multiple products competing as Bertrand oligopolists with slightly differentiated goods. The quantity vector of each firm *i* is given by *Q*_*i*_(*P*), where *P* is the vector of all prices in the industry and *P*_*i*_ is the component of *P* with prices for goods of firm *i*. Profit for firm *i* is given by $\pi_{i}=P_{i}^{⊺}Q_{i}\left(P\right)-C_{i}\left(Q_{i}\left(P\right)\right)$, where *C*_*i*_ is the cost function for firm *i*.

The standard UPP formulation is as follows. Suppose firms *i* and *j* merge. The profit maximization problem for the merged firm is given by

$$\begin{array}{c} \text{max}\Pi_{M}=P_{i}^{⊺}Q_{i}\left(P\right)+P_{j}^{⊺}Q_{j}\left(P\right)-C_{i}\left(Q_{i}\left(P\right)\right)-C_{j}\left(Q_{j}\left(P\right)\right) \end{array}$$

The first order condition (with respect to *P*_*i*_) may be written as:

$$\begin{array}{c} \begin{array}{cc} h_{i}\left(P\right) & \equiv -(\frac{\partial Q_{i}{\left(P\right)}^{⊺}}{\partial P_{i}}{)}^{-1}Q_{i}\left(P\right)-(P_{i}-\frac{\partial C_{i}}{\partial Q_{i}\left(P\right)})\\ & +(\frac{\partial Q_{i}{\left(P\right)}^{⊺}}{\partial P_{i}}{)}^{-1}(\frac{\partial Q_{j}{\left(P\right)}^{⊺}}{\partial P_{i}})(P_{j}-\frac{\partial C_{j}}{\partial Q_{j}\left(P\right)})=0 \end{array} \end{array}$$

Comparing this to the first-order condition for firm *i* pre-merger yields upward pricing pressure for good *i*.

$$\begin{array}{c} UPP_{i}=\underset{D_{ij}}{\underset{︸}{-(\frac{\partial Q_{i}{\left(P\right)}^{⊺}}{\partial P_{i}}{)}^{-1}(\frac{\partial Q_{j}{\left(P\right)}^{⊺}}{\partial P_{i}})}}\underset{\left(P_{j}-MC_{j}\right)}{\underset{︸}{\left(P_{j}-\frac{\partial C_{j}}{\partial Q_{j}}\right)}} \end{array}$$

This is the standard UPP formulation used widely in the literature and in antitrust practice. The term—(*∂Q*_*i*_(*P*)^⊺^/*∂P*_*i*_)^−1^ (*∂Q*_*j*_(*P*)^⊺^/*∂P*_*i*_) is the diversion matrix, which measures proportion of sales lost by firm *i* that are recaptured by firm *j*, and (*P*_*j*_ − *∂C*_*j*_/*∂Q*_*j*_) is the margin for firm *j*. Both are evaluated at pre-merger values.

Notice that in this formulation there are no merger-specific cost efficiencies, because total cost for the merged firm is the sum of costs of the merging partners and there are no cross-firm cost complementarities. In order to distinguish this from other UPP calculations, we shall denote this standard formulation with no efficiencies as *UPP*_*NoEff*_.

We include cross-firm cost complementarities by adding an interactive term in the profit-maximization problem of the merged firm as follows.

$$\begin{array}{c} \text{max}\Pi_{M}=P_{i}^{⊺}Q_{i}\left(P\right)+P_{j}^{⊺}Q_{j}\left(P\right)-\left[C_{i}\left(Q_{i}\left(P\right)\right)+C_{j}\left(Q_{j}\left(P\right)\right)\;-\;\phi \left(Q_{i}\left(P\right),Q_{j}\left(P\right)\right)\right] \end{array}$$

The term *ϕ*(*Q*_*i*_(*P*), *Q*_*j*_(*P*)) is an adjustment (reduction) to total cost of the merged firm that depends on output of both firms. In order to capture merger-specific efficiencies, we require this term to be zero if output of either firm is zero: *ϕ*(*Q*_*i*_(*P*), 0)) = *ϕ*(0, *Q*_*j*_(*P*)) = 0.

The first-order condition for this problem is given by

$$\begin{array}{c} \begin{array}{c} {\tilde{h}}_{i}\left(P\right)=-(\frac{\partial Q_{i}{\left(P\right)}^{T}}{\partial P_{i}}{)}^{-1}Q_{i}\left(P\right)-(P_{i}-\frac{\partial C_{i}}{\partial Q_{i}\left(P\right)}+\frac{\partial \phi (Q_{i}\left(P\right),Q_{j}\left(P\right))}{\partial Q_{i}\left(P\right)})\\ -(\frac{\partial Q_{i}{\left(P\right)}^{T}}{\partial P_{i}}{)}^{-1}(\frac{\partial Q_{j}{\left(P\right)}^{T}}{\partial P_{i}})(P_{j}-\frac{\partial C_{j}}{\partial Q_{j}\left(P\right)}+\frac{\partial \phi (Q_{i}\left(P\right),Q_{j}\left(P\right))}{\partial Q_{j}\left(P\right)}) \end{array} \end{array}$$ (1)

Comparing this to the pre-merger first-order condition yields the following new UPP formulation.

$$\begin{array}{c} {\tilde{UPP}}_{i}\left(P\right)=D_{ij}\left(P_{j}-MC_{j}\right)-\underset{\text{Efficiency}\;i}{\underset{︸}{\left(\frac{\partial \phi (Q_{i}\left(P\right),Q_{j}\left(P\right))}{\partial Q_{i}\left(P\right)}\right)}}+D_{ij}\underset{\text{Efficiency}\;j}{\underset{︸}{\left(\frac{\partial \phi (Q_{i}\left(P\right),Q_{j}\left(P\right))}{\partial Q_{j}\left(P\right)}\right)}} \end{array}$$ (2)

The general form of this formulation exists in the literature, as shown in [17], [1], and [2]. In the more specific formulation used here, efficiencies show up in a tractable and intuitive manner. The term *D*_*ij*_(*P*_*j*_ − *MC*_*j*_) is the standard UPP formulation. The term $\partial \phi (Q_{i}(P),Q_{j}(P)){/}_{\partial Q_{i}(P)}$ may be viewed as marginal, merger-specific own firm efficiency. It is an adjustment to the standard UPP formulation arising from own firm efficiency and it serves to lower upward pricing pressure for good *i*. The term $\partial \phi (Q_{i}(P),Q_{j}(P)){/}_{\partial Q_{j}(P)}$ is marginal, merger-specific partner firm efficiency. It is an adjustment to the standard UPP formulation arising from partner firm efficiency (modified by the diversion matrix) and it serves to increase upward pricing pressure for good *i*. The UPP formulation with efficiencies adjusts the standard UPP formulation for both these effects. In order to distinguish this from other UPP calculations, we shall denote this formulation with merger-specific efficiencies as *UPP*_*ModEff*_.

As is well-known, the standard UPP formulation does not capture the full first-order effect for a merged firm to raise prices. As shown in the literature, in order to get an accurate first order approximation of the impulse to raise prices post-merger, the UPP calculation should be modified by the post-merger pass through matrix. This translates into the following UPP formulation with first-order approximation.

$$\begin{array}{c} UPP_{FOA}=-(\frac{\partial \tilde{h}}{\partial P}\left(P^{0}\right){)}^{-1}\tilde{UPP} \end{array}$$ (3)

Here, $\tilde{h}$ is the first-order condition (listed above) for the merged firm and $(\partial \tilde{h}/\partial P\left(P^{0}\right){)}^{-1}$ and $\tilde{UPP}$ are evaluated at pre-merger equilibrium prices. *UPP*_*FOA*_ uses a theoretically accurate measure of the change in best response of the merged firm as compared to the firm pre-merger.

The next section implements these formulations in a Monte Carlo setting.

## Monte Carlo

In order to estimate the effect of the theoretical framework with cost efficiencies on the post-merger equilibrium and different measures of UPP, we use different economic environments to simulate the model. We use four different demand formulations and two different cost formulations for a total of eight different scenarios.

For the demand side, we use four standard functional forms that have been used widely in academic research and merger analysis [12, 14, 35]. These are Logit demand, Log-Linear demand, Linear demand, and Almost Ideal demand. These are also used in other Monte Carlo studies of UPP [20, 23, 26]. Our demand calibration strategy follows [26], as described in detail in their appendix (We are grateful to Professor Nathan Miller for sharing his code for this calibration).

For the cost side, we use two functional forms used in the existing literature: Generalized Leontief cost [36] and Quadratic cost [37, 38].

The multiple good Generalized Leontief formulation is the following [39–41]:

$$\begin{array}{c} C\left(Q\right)=\sum_{i}^{m}\sum_{j}^{m}\alpha_{ij}{\left(Q_{i}Q_{j}\right)}^{\frac{1}{2}} \end{array}$$

In the special case when firms *i* and *j* merge, and each firm produces one good, the cost function for the merged firm is given by:

$$\begin{array}{c} C\left(Q_{i},Q_{j}\right)=\alpha_{ii}Q_{i}+\alpha_{jj}Q_{j}-\alpha_{ij}Q_{i}^{½}Q_{j}^{½} \end{array}$$

In this case, the interactive term is $\phi \left(Q_{i},Q_{j}\right)=\alpha_{ij}Q_{i}^{½}Q_{j}^{½}$ and it satisfies merger-specific cross complementarity that cannot be realized apart from consolidation; *ϕ*(*Q*_*i*_, 0) = *ϕ*(0, *Q*_*j*_) = 0. Notice that

$$\begin{array}{l} \frac{\partial \phi \left(Q_{i},Q_{j}\right)}{\partial Q_{i}}=0.5\alpha_{ij}(\frac{Q_{j}}{Q_{i}}{)}^{½}\\ \frac{\partial \phi \left(Q_{i},Q_{j}\right)}{\partial Q_{j}}=0.5\alpha_{ij}(\frac{Q_{i}}{Q_{j}}{)}^{½} \end{array}$$

The multiple good Quadratic formulation is the following [41]:

$$\begin{array}{c} C\left(Q\right)=\sum_{i}^{m}Q_{i}^{2}+\frac{1}{2}\sum_{i}^{m}\sum_{j\neq i}^{m}\beta_{ii}Q_{i}Q_{j} \end{array}$$

In the special case when firms *i* and *j* merge, and each firm produces one good, the cost function for the merged firm is given by:

$$\begin{array}{c} C\left(Q_{i},Q_{j}\right)=\beta_{ii}Q_{i}^{2}+\beta_{jj}Q_{j}^{2}-\beta_{ij}Q_{i}Q_{j} \end{array}$$

In this case, the interactive term is *ϕ*(*Q*_*i*_, *Q*_*j*_) = *β*_*ij*_*Q*_*i*_*Q*_*j*_ and it also satisfies merger-specific cross complementarity that is activated only from a merger, in the sense that *ϕ*(*Q*_*i*_, 0) = *ϕ*(0, *Q*_*j*_) = 0. Notice that

$$\begin{array}{c} \begin{array}{l} \frac{\partial \phi \left(Q_{i},Q_{j}\right)}{\partial Q_{i}}=\beta_{ij}Q_{j}\\ \frac{\partial \phi \left(Q_{i},Q_{j}\right)}{\partial Q_{j}}=\beta_{ij}Q_{i} \end{array} \end{array}$$

The data generating process is the following.

We suppose that each industry contains four firms competing in prices with differentiated goods. Each firm produces a single output and industry equilibrium is Bertrand-Nash.

1. Market shares are randomly drawn for each of the four firms and an outside good. The actual market shares that are used in the process are normalized to aggregate to one for the market in question. The margin for the first firm is randomly drawn with support [0.2, 0.8].
2. The parameters for the interactive term in the cost structures are randomly drawn with support [0, 1]. The rationale behind the support of these parameters being non-negative is as follows: If the firms would be more inefficient operating jointly than separately, then even if they merge, there is reason enough to believe they would continue operations disjointly.
3. Given the market shares and margins, it is possible to calibrate a Logit demand system, and thus, demand elasticities in the pre-merger equilibrium. Notice that the demand system is such that its parameters are chosen to rationalize the data drawn in the previous steps. In this study, consumer substitution behavior is proportional to market shares. These parameters are identified exactly given market shares, prices, and a single margin.
4. Once the Logit demand system is obtained, it is possible to calibrate the remaining demand functional forms (Log-Linear, Linear and Almost Ideal) such that they are compatible with the Logit demand elasticities. Similarly to the Logit case, the demand systems’ parameters are perfectly identified given market shares, prices, and Logit demand elasticities.
5. In each draw, two firms go through a merger. Post-merger equilibrium prices are computed as well as various measures of upward pricing pressure and first order approximation.
6. Repeat these steps until 5,000 draws of data are obtained.

This process yields a total of 40,000 mergers (8 scenarios with 5,000 mergers each).

In order to analyze the accuracy of UPP for price prediction and for merger screening, we use the following four measures.

- *UPP*_*NoEff*_—This is the standard and widely used UPP calculation with no efficiencies. It serves as a baseline.
- *UPP*_*AvgEff*_—This is the standard UPP calculation adjusted for average merger efficiencies. It serves as a benchmark for current practice.
- *UPP*_*ModEff*_—This is UPP with merger-specific cost efficiencies, as derived above and as discussed in more detail below.
- *UPP*_*FOA*_—This is UPP with merger-specific efficiencies and first-order approximation, as derived above and as discussed in more detail below.

A starting point for UPP calculations is the standard UPP calculation measuring the value of diverted sales.

$$\begin{array}{c} UPP_{NoEff}=UPP_{i}=D_{ij}\left(P_{j}-MC_{j}\right) \end{array}$$ (4)

As discussed above, *UPP*_*NoEff*_ does not include cost efficiencies. This serves as a baseline for additional analysis.

The second measure we use is the value of diverted sales adjusted for average merger efficiencies.

$$\begin{array}{c} UPP_{AvgEff}=D_{ij}\left(P_{j}-MC_{j}\right)\underset{Both}{\underset{︸}{-\underset{Own}{\underset{︸}{\bar{\left(\frac{\partial \phi }{\partial Q_{i}}\right)}}}+D_{ij}\bar{\left(\frac{\partial \phi }{\partial Q_{j}}\right)}}} \end{array}$$ (5)

This measure is an estimate for current practice in the following sense. It is well-known that in the absence of cost efficiencies, UPP tends to overestimate the increase in post-merger prices. The standard current practice to account for this is to lower the UPP computation by some amount, motivating it as a reduction due to cost efficiencies. The amount of this reduction is a frequent source of debate. When UPP computation is high, merging parties argue with antitrust agencies that the UPP calculation should be lowered significantly, because there are cost efficiencies from the merger, but antitrust agencies argue for more realistic numbers and require additional justification. Current practice is to arrive at some adjustment, in the form of an efficiency credit.

The measure *UPP*_*AvgEff*_ is a benchmark for the current practice of efficiency credits, in the sense that it adjusts baseline UPP calculation *UPP*_*NoEff*_ for the average efficiency realized under a particular cost complementarity structure. In other words, in the absence of modeling cost efficiencies, if merging parties and antitrust agencies have to agree to an efficiency credit, their best guess would be the efficiency that a particular technology generates on average, yielding the measure *UPP*_*AvgEff*_.

The third measure we use is the UPP calculation adjusted for merger-specific cost efficiencies. This is what the standard UPP calculation would be if we derived it using the model above.

$$\begin{array}{c} UPP_{ModEff}=D_{ij}\left(P_{j}-MC_{j}\right)\underset{Both}{\underset{︸}{-\underset{Own}{\underset{︸}{\left(\frac{\partial \phi }{\partial Q_{i}}\right)}}+D_{ij}\left(\frac{\partial \phi }{\partial Q_{j}}\right)}} \end{array}$$ (6)

Notice that the only additional information needed to implement *UPP*_*ModEff*_ as compared to *UPP*_*NoEff*_ is the change in total cost due to marginal merger-specific efficiencies (In particular, a change in fixed cost due to a merger does not affect these calculations). In other words, adding a fixed cost term in the total cost curve does not affect the first-order conditions and therefore, does not affect calculations based on changes in the first-order conditions. In this sense, the first-order approach to merger analysis (and consequently, derivations based on it, such as UPP) does not automatically include changes in fixed costs due to a merger. When fixed cost savings are important, these should be included as additional information in merger evaluation and would be useful in a more detailed review of the merger. Merging firms typically provide this type of information to regulators as supporting information for adjustments to UPP calculations. The formulation here shows how this information and the diversion matrix can be used by practitioners to derive a better estimate. Notably, in order to implement the formulation here, the full functional form of the cost curve is not needed; the additional information needed is marginal merger-specific efficiencies.

The final measure we use adjusts the UPP computation with cost efficiencies by the pass-through matrix.

$$UPP_{FOA}=-{\left(\frac{\partial \tilde{h}}{\partial P}\left(P^{0}\right)\right)}^{-1}\left(D_{ij}\left(P_{j}-MC_{j}\right)\underset{Both}{\underset{︸}{-\underset{Own}{\underset{︸}{\left(\frac{\partial \phi }{\partial Q_{i}}\right)}}+D_{ij}\left(\frac{\partial \phi }{\partial Q_{j}}\right)}}\right)$$ (7)

Here, $\tilde{h}$ is the first-order condition for the merged firm and $(\partial \tilde{h}/\partial P\left(P^{0}\right){)}^{-1}$ is the post-merger pass-through matrix. We know from [26] that in the absence of merger-specific efficiencies, pass-through matrix depends on first and second derivative of demand and does not require higher order information. Inspection of the form of $\tilde{h}$ with merger-specific efficiencies (see Eq 1) shows that with efficiencies, the additional information needed to implement *UPP*_*FOA*_ is the first and second derivatives of merger-specific efficiencies. Higher order information or the full functional form of merger-specific efficiencies are not needed. Moreover, the inverse of the Jacobian of $\tilde{h}$ used in *UPP*_*FOA*_ is typically computed numerically as analytic forms are not available. Several statistical packages are available in this regard. For example, consider [42] for a package in R and [43] for STATA.

For each merger, we compute these four UPP measures and compare them with the post-merger equilibrium price.

The next section presents the results of the Monte Carlo simulations.

## Results

Table 1 presents some descriptive summary statistics for the data generated using Monte Carlo. The median market share for firms is 20%, which is consistent with drawing market shares for four firms and an outside good. Eighty percent of the margins are distributed between 0.247 and 0.746 with median at 0.471 (these are pre-merger margin values, not including merger efficiencies).

**Table 1:** Descriptive statistics.

	Median	10%	25%	75%	90%
Market Conditions					
Market share	0.201	0.050	0.114	0.276	0.339
Margin	0.471	0.247	0.333	0.623	0.746
Market Concentration					
Pre-Merger	1981	1418	1642	2436	2872
Post-Merger	2706	1795	2159	3360	4066
Δ*HHI*	654	113	310	1075	1527
Upward Pricing Pressure					
*UPP*_*NoEff*_	0.107	0.021	0.053	0.184	0.265
*UPP*_*AvgEff*_	0.021	-0.115	-0.045	0.104	0.192
*UPP*_*ModEff*_	0.013	-0.248	-0.070	0.058	0.108
*UPP*_*FOA*_	0.012	-0.619	-0.115	0.074	0.253
Own Merger Pass-Through					
Logit	0.801	0.675	0.733	0.880	0.943
Linear	0.533	0.510	0.520	0.550	0.569
Log-Linear	1.927	1.344	1.515	2.671	3.601
Almost Ideal	1.210	0.782	0.907	1.776	2.522
Cross Merger Pass-Through					
Logit	0.038	0.007	0.018	0.063	0.089
Linear	0.090	0.024	0.053	0.120	0.152
Log-Linear	0.000	-0.000	-0.000	0.000	0.000
Almost Ideal	0.227	0.057	0.125	0.409	0.778
Merger Price Effects					
Logit	0.023	-0.158	-0.019	0.064	0.113
Linear	0.017	-0.137	-0.038	0.054	0.106
Log-Linear	0.078	-0.444	-0.047	0.288	0.939
Almost Ideal	0.050	-0.287	-0.025	0.175	0.503

*Notes*: Summary statistics are based on 5,000 randomly-drawn sets of data on the pre and post-merger equilibria. The values for market share and margin are for all four firms. Market share and margin are drawn randomly in the data generating process. Own merger pass-through is the first element of the diagonal of ${\left[\partial \tilde{h}\left(P\right)/\partial P\left(P^{0}\right)\right]}^{-1}$, and cross merger pass-through is the first off-diagonal element of ${\left[\partial \tilde{h}\left(P\right)/\partial P\left(P^{0}\right)\right]}^{-1}$. The merger price effects are the change in firm 1’s equilibrium price.

**Fig 1:**
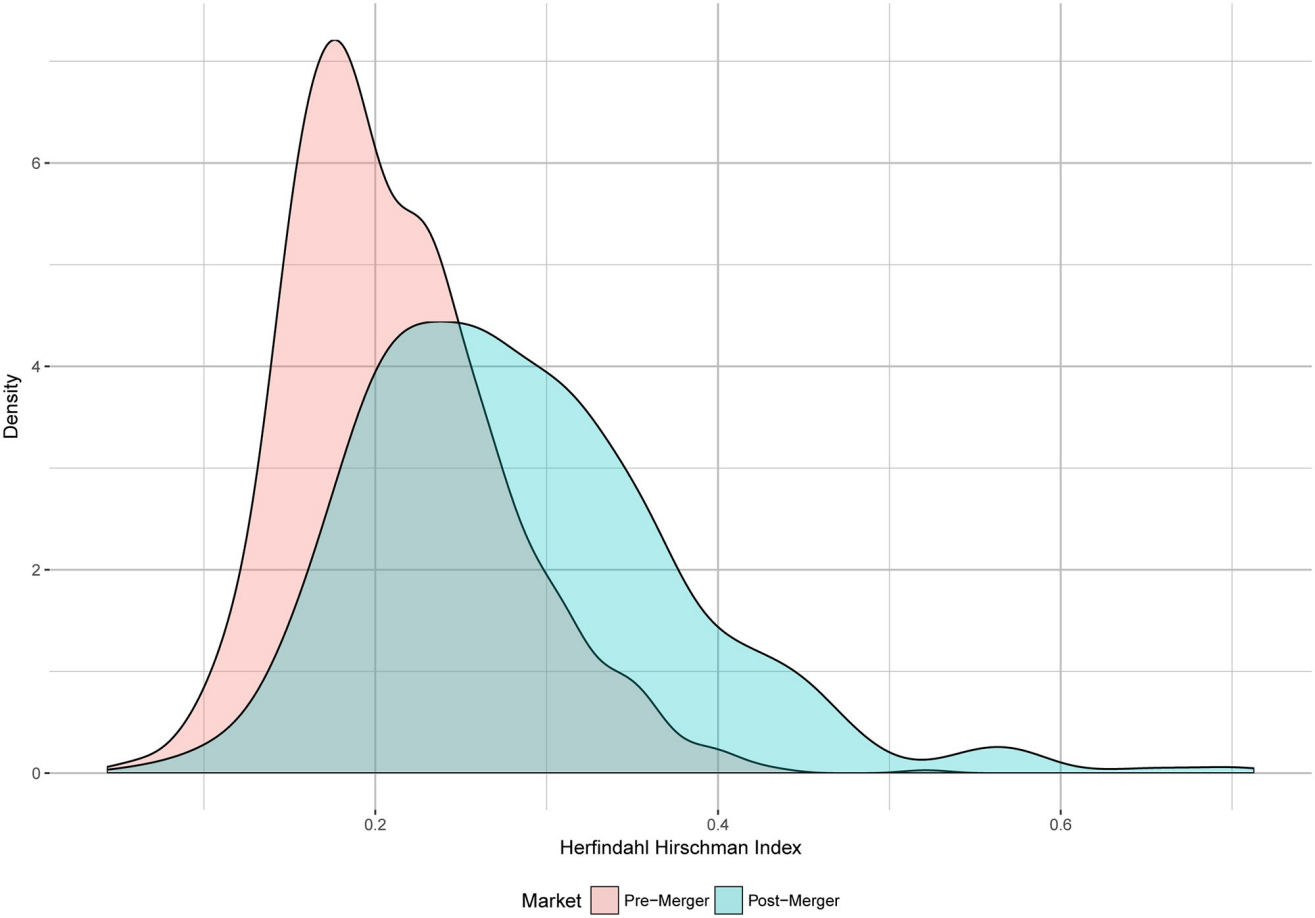
Market concentration pre and post-merger. Shows the distribution of market concentration within the markets pre and post merger as measured by the Herfindahl Hirschman Index. Simplifying assumption for post-merger increase in HHI is twice the product of the merging parties pre-merger market share, as used in merger screening.

Market concentration, as measured by HHI, has a median pre-merger value of 1981, considered to be a moderately concentrated market according to the Horizontal Merger Guidelines (2010). According to the U.S. Department of Justice and Federal Trade Commission Horizontal Merger Guidelines (2010), §5, a market is considered unconcentrated if *HHI* ≤ 1500, moderately concentrated if 1500 < *HHI* ≤ 2500 and highly concentrated if *HHI* > 2500. Already at the 10^th^ percentile, markets are at least moderately concentrated, whereas at the 90^th^ percentile markets are highly concentrated pre-merger. This is consistent with a market comprised of four firms—a market with four equal sized firms would be on the threshold between moderately and highly concentrated. Market concentration post-merger, as measured by HHI has a median of 2706, a highly concentrated market. Eighty percent of the markets are between 1795 and 4066. This increase in concentration is consistent with a market reduced to three firms. Δ*HHI* has median of 654, which would trigger further scrutiny from the Agencies—according to HMG (2010) §5, mergers that increase HHI by less than 100 are unlikely to be challenged, whereas mergers that increase it by more than 200 will likely require further action. Fig 1 shows the density kernels for HHI’s pre- and post-merger for all mergers.

Different measures of UPP yield different post-merger price predictions. *UPP*_*NoEff*_ results in a 10.7% increase in prices at the median, with eighty percent distributed between 2.1% and 27.5%. With more accurate methods to incorporate merger specific efficiencies, there are lower increases in price prediction. For *UPP*_*FOA*_, it is 1.2% and eighty percent distributed between −61.9% and 25.3%.

Own merger pass through is highest with Log-Linear demand and lowest with Linear demand. Cross-merger pass-through is highest with Almost Ideal demand and lowest with Log-Linear demand. Merger price effects are smallest with Linear demand (1.7% at the median), then Logit demand (2.7%), then Almost Ideal demand (5%), and then Log-Linear demand (7.8%).

### Price prediction accuracy

For price prediction accuracy, we compute absolute errors and relative errors as follows.

Absolute prediction error: *APE* = |*P*_*UPP*_ − *P*_*Post*_|
Relative prediction error: RPE=|PUPP−PPost|PPost

Here *P*_*UPP*_ is the price given by a particular UPP calculation and *P*_*Post*_ is the computed post-merger equilibrium price. As pre-merger prices are normalized to unity, APE gives prediction error in percentage points and RPE gives percent error. For example, if *P*_*UPP*_ = 1.11 and *P*_*Post*_ = 1.05 then (because pre-merger price is 1), *APE* = 6 percentage points and *RPE* = 5.7 percent.

**Fig 2:**
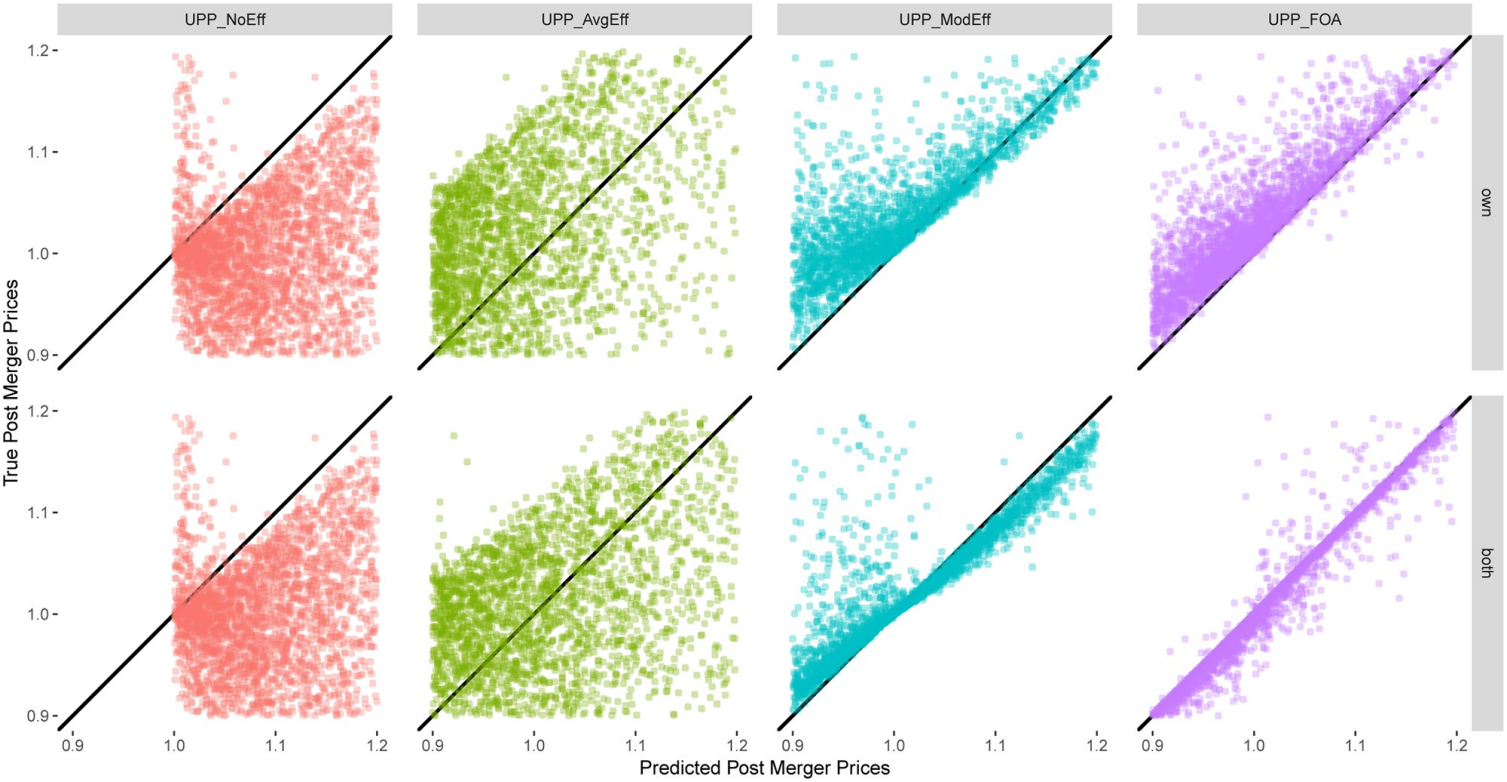
Accuracy of prediction—Logit demand, Generalized Leontief cost. First row shows the distribution of the true post merger prices against the predicted post merger prices using different UPP calculations and own goods’ efficiencies included in the computation. Second row shows the same for both goods’ efficiencies.

Fig 2 presents the analysis for the environment with Logit demand system and Generalized Leontief cost structure. The figure has eight panels. The four columns correspond to the four UPP calculations defined above: *UPP*_*NoEff*_, *UPP*_*AvgEff*_, *UPP*_*ModEff*_, and *UPP*_*FOA*_. The top row corresponds to the case where these calculations are made using own firm efficiency only (a case that is frequently used in practice) and the bottom row corresponds to the case where these calculations are made using both own firm efficiency and partner firm efficiency. The bottom row uses the four calculations defined above.

In each panel, the x-axis measures the predicted post-merger price using a particular UPP calculation, and the y-axis measures the true post-merger equilibrium price. Each point in a panel corresponds to one merger. Points on the diagonal are those mergers for which the price prediction using the UPP calculation for that panel is exactly the same as the true post-merger equilibrium price. Points above the diagonal are those mergers for which the UPP calculation under-predicts the true post-merger price. Points below the diagonal are those mergers for which the UPP calculation over-predicts the true post-merger price.

**Fig 3:**
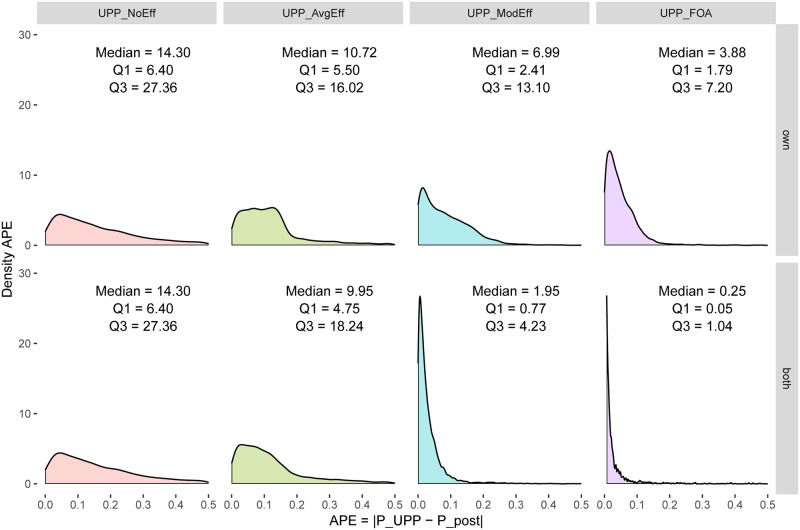
Absolute prediction errors—Logit demand system, Generalized Leontief cost. Portrays density kernels for absolute prediction errors, as well as the median absolute prediction error, first and third quartile for each specification.

**Fig 4:**
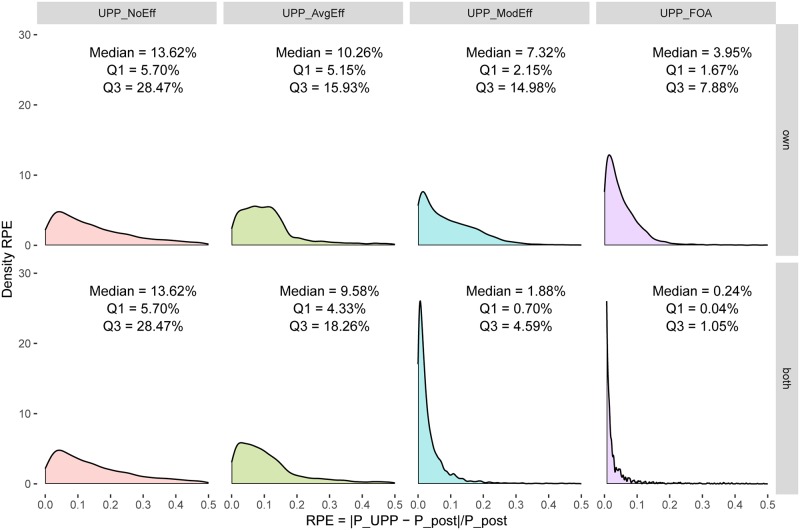
Relative prediction errors—Logit demand system, Generalized Leontief cost. Portrays density kernels for relative prediction errors, as well as the median relative prediction error, first and third quartile for each specification.

Consider the first column in Fig 2. In the top panel (labeled *UPP*_*NoEff*_), the x-axis measures the post-merger price increase using *UPP*_*NoEff*_ and the y-axis measures the true post-merger equilibrium price. As *UPP*_*NoEff*_ excludes efficiencies, most of the data over-predicts the true post-merger prices and lies below the diagonal, as expected. The data appear truncated at 1.0 (the pre-merger equilibrium price) because in the absence of cost efficiencies, *UPP*_*NoEff*_ predicts a price increase, even when the true post-merger price is lower, as expected. The bottom panel is the same as the top panel, because the difference in UPP calculation between own firm efficiency and the combined efficiency of both firms arises only when the UPP calculation includes efficiencies. In both panels, median APE is 14.3 p.p. and median RPE is 13.6%. The density kernels of APE are given in the corresponding panels in Fig 3 and that of RPE in the corresponding panels in Fig 4.

The second column in Fig 2 (labeled *UPP*_*AvgEff*_) adjusts *UPP*_*NoEff*_ for an efficiency credit based on the average efficiency generated by a particular technology (Generalized Leontief in this case). As discussed above, this a proxy for the current practice of adjusting the UPP calculation for an efficiency credit. As compared to panels in column 1, this moves the data toward the left. The top panel in this column considers average efficiency for own firm only and the bottom panel considers average combined efficiency for both partners in a merger. As compared to the first column, the data in the second column is dispersed somewhat more evenly across the diagonal, indicating improved price prediction accuracy. This shows up in lower price prediction errors. In the top panel, median APE is 10.7 p.p. (a gain in price prediction accuracy of about 3.6 percentage points over *UPP*_*NoEff*_) and median RPE is 10.3% (a gain of about 3.3 percentage points over *UPP*_*NoEff*_). In the bottom panel, the corresponding numbers are 9.9 p.p. for APE (a gain of 4.4 p.p.) and 9.6% for RPE (a gain of 4 p.p.). The density kernels of APE are given in the corresponding panels in Fig 3 and that of RPE in the corresponding panels in Fig 4.

The third column in Fig 2 (labeled *UPP*_*ModEff*_) uses the new UPP calculation based on model-based, merger-specific Generalized Leontief cost complementarities. Both panels show a noticeable clustering of the data around the diagonal, indicating further improvements in price prediction. In the top panel, median APE is 7 p.p. (a gain of 3.7 percentage points over current practice proxy using *UPP*_*AvgEff*_) and median RPE is 7.3% (a gain of about 3.3 percentage points over *UPP*_*AvgEff*_). The bottom panel shows that accounting for the combined efficiency effect of both merger partners yields even greater gains in price prediction accuracy, consistent with the theory. In the bottom panel, median APE is 2 p.p. (a gain of 7.9 p.p. over *UPP*_*AvgEff*_) and median RPE is 1.9% (a gain of 7.7 p.p.). The density kernels of APE are given in the corresponding panels in Fig 3 and those of RPE in the corresponding panels in Fig 4.

Finally, the fourth column in Fig 2 (labeled *UPP*_*FOA*_) uses the first-order approximation to adjust *UPP*_*ModEff*_ by the pass-through matrix. As mentioned above, this is a theoretically accurate measure of the first impulse to change prices. Both panels show greater clustering of data around the diagonal, with notable improvement in the bottom panel. In the top panel, median APE shrinks to 3.9 p.p. (a gain of 6.8 percentage points over current practice proxy using *UPP*_*AvgEff*_) and median RPE is 4% (a gain of about 7.3 percentage points over *UPP*_*AvgEff*_). In the bottom panel, median APE is only 0.3 p.p. (a gain of 9.6 p.p. over *UPP*_*AvgEff*_) and median RPE is 0.2% (a gain of 9.4 p.p.).

Put differently, in the bottom panel of column four, absolute price prediction errors decrease 97% (from 9.9 p.p. to 0.3 p.p., at the median) and relative price prediction errors decrease 97% (from 9.6% to 0.2%, at the median) as we move from current practice (using *UPP*_*AvgEff*_) to a more theoretically accurate measure using *UPP*_*FOA*_. More generally, the entire density kernel of the corresponding APE (Fig 3, bottom right panel) and of RPE (Fig 4, bottom right panel) compresses toward zero.

Figs 2–4 indicate presence of substantial gains from reforming the standard UPP calculation to include cost efficiencies (for both merging partners) and in a manner guided by the model and to use first-order approximation. These results are based on Logit demand and Generalized Leontief costs. A similar pattern is seen for the other seven scenarios as well. This is documented in S1–S21 Figs.

A summary of all eight scenarios is given in Table 2. As shown in Table 2, in seven of eight scenarios, *UPP*_*FOA*_ based price predictions are within 3% of post-merger equilibrium prices at the median, both in absolute and relative terms.

**Table 2:** Improvement in price prediction.

Generalized Leontief cost									
Logit Demand	NoEff	AvgEff	ModEff	FOA	Linear Demand	NoEff	AvgEff	ModEff	FOA
	APE (p.p.)					APE (p.p.)			
Median	14.30	9.95	1.95	0.25	Median	14.34	8.73	2.26	0.00
Absolute Gain over AvgEff			8.00	9.69	Absolute Gain over AvgEff			6.47	8.73
Relative Gain over AvgEff			80.39	97.47	Relative Gain over AvgEff			74.08	100.00
	RPE (%)					RPE (%)			
Median	13.62	9.58	1.88	0.24	Median	14.38	8.72	2.23	0.00
Absolute Gain over AvgEff			7.70	9.34	Absolute Gain over AvgEff			6.50	8.72
Relative Gain over AvgEff			80.33	97.51	Relative Gain over AvgEff			74.49	100.00
Log-Linear Demand	NoEff	AvgEff	ModEff	FOA	Almost Ideal Demand	NoEff	AvgEff	ModEff	FOA
	APE (p.p.)					APE (p.p.)			
Median	33.15	27.79	19.55	15.18	Median	21.43	16.71	8.17	1.71
Absolute Gain over AvgEff			8.24	12.60	Absolute Gain over AvgEff			8.54	14.99
Relative Gain over AvgEff			29.64	45.36	Relative Gain over AvgEff			51.10	89.74
	RPE (%)					RPE (%)			
Median	34.75	28.79	22.92	16.04	Median	22.01	16.93	9.15	1.76
Absolute Gain over AvgEff			5.87	12.75	Absolute Gain over AvgEff			7.78	15.17
Relative Gain over AvgEff			20.40	44.27	Relative Gain over AvgEff			45.94	89.60
Quadratic Cost									
Logit Demand	NoEff	AvgEff	ModEff	FOA	Linear Demand	NoEff	AvgEff	ModEff	FOA
	APE (p.p.)					APE (p.p.)			
Median	5.89	3.84	0.62	0.02	Median	6.15	3.94	1.12	0.00
Absolute Gain over AvgEff			3.22	3.82	Absolute Gain over AvgEff			2.83	3.94
Relative Gain over AvgEff			83.81	99.47	Relative Gain over AvgEff			71.67	100.00
	RPE (%)					RPE (%)			
Median	5.63	3.73	0.59	0.02	Median	5.91	3.82	1.08	0.00
Absolute Gain over AvgEff			3.14	3.71	Absolute Gain over AvgEff			2.75	3.82
Relative Gain over AvgEff			84.16	99.49	Relative Gain over AvgEff			71.86	100.00
Log-Linear Demand	NoEff	AvgEff	ModEff	FOA	Almost Ideal Demand	NoEff	AvgEff	ModEff	FOA
	APE (p.p.)					APE (p.p.)			
Median	5.37	8.73	9.60	2.92	Median	2.61	4.30	3.60	0.21
Absolute Gain over AvgEff			-0.87	5.81	Absolute Gain over AvgEff			0.70	4.08
Relative Gain over AvgEff			-9.98	66.54	Relative Gain over AvgEff			16.27	95.00
	RPE (%)					RPE (%)			
Median	4.71	7.68	8.32	2.29	Median	2.36	4.11	3.33	0.20
Absolute Gain over AvgEff			-0.64	5.39	Absolute Gain over AvgEff			0.78	3.91
Relative Gain over AvgEff			-8.38	70.15	Relative Gain over AvgEff			18.92	95.21

The log-linear case is an exception, likely related to curvature of utility, causing the diagonal elements of the merger pass-through matrix to exceed one, as documented in [26]. The average reduction in price prediction errors (*UPP*_*FOA*_ compared to *UPP*_*AvgEff*_) in these seven scenarios is 93%.

Moreover, in five scenarios, *UPP*_*FOA*_ based price predictions are within 0.25% of post-merger equilibrium prices at the median, both in absolute and relative terms. The average reduction in price prediction errors (*UPP*_*FOA*_ compared to *UPP*_*AvgEff*_) in these five scenarios is 98%. Altogether, the results show considerable evidence for using cost efficiencies in the manner guided by the model and a more accurate first-order approximation in UPP calculations.

### Merger screening accuracy

We also use these data to investigate accuracy of different UPP formulations as pre-merger screening tools. As mentioned earlier, UPP is being used increasingly as a pre-merger screening tool by antitrust agencies in the United States and worldwide, mainly because it is relatively quick and easy to implement, requires less information than some other measures, and is grounded in theory.

The typical use of UPP is to flag a merger for further scrutiny if the UPP calculation is above a given threshold. As UPP is not a perfect predictor of post-merger prices, this leads to two familiar errors: false positives and false negatives.

A false positive occurs when the UPP screen flags a merger for further analysis but post-merger equilibrium prices are below the acceptable threshold. A false positive may lead to unnecessary use of resources by both the antitrust agencies and the merging parties to investigate or block a merger that does not have significant anticompetitive effects. We term this a Type I error.

A false negative occurs when the UPP screen does not flag a merger for further analysis but post-merger equilibrium prices are above the acceptable threshold. A false negative allows a merger to go through even if it has significant anticompetitive effects and may harm consumers. We term this a Type II error.

As a baseline, consider a 5% price increase threshold. This is a common threshold in antitrust analysis, and is also used in the SSNIP test.

**Fig 5:**
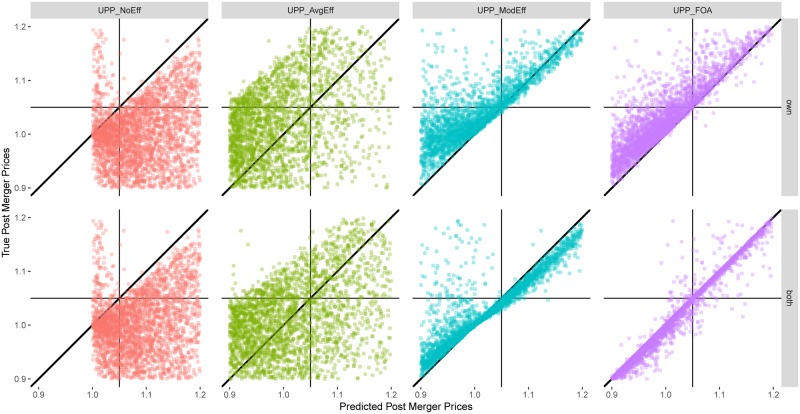
5% threshold on decision rule for price increases.

Graphically, in each panel in Fig 5, draw a vertical line intersecting the x-axis at 1.05, and a horizontal line intersecting the y-axis at 1.05. Mergers to the right of the vertical line are flagged for further scrutiny by the UPP screen and mergers to the left are not. Mergers above the horizontal line have high post-merger price increases (relative to the acceptable threshold) and mergers below the line have low post-merger price increases. Therefore, all mergers in the bottom right quadrant are false positives (flagged for further scrutiny incorrectly) and all mergers in the top left quadrant are false negatives (letting a merger go through incorrectly).

As expected, and as shown in the first column in Fig 5 (labeled *UPP*_*NoEff*_), in the presence of merger efficiencies, not including these efficiencies in UPP calculation leads to a sizable number of false positives (about 57.9 percent of all mergers) and perhaps a few false negatives (3.5 percent). In this case, the total probability of making a type I or type II error is 0.614 (about 61.4 percent of all mergers).

Adjusting UPP for average efficiencies for both merger partners (second column, lower row in Fig 5), the probability of false positives declines to 0.175, probability of false negatives increases to 0.092, and total probability of type I and type II error decreases to 0.267. This is what may be expected using the current practice of efficiency credits (in the scenario with Logit demand and Generalized Leontief cost complementarities).

Using the *UPP*_*ModEff*_ calculation that includes model-based cost efficiencies (third column, lower row in Fig 5), the total probability of making a type I or type II error goes down to 0.057, and using *UPP*_*FOA*_ calculation lowers this total probability even more to 0.017 (about 1.7 percent of all mergers).

In other words, total probability of making a merger screening error decreases 79% (from 0.267 to 0.057) as we move from current practice (using *UPP*_*AvgEff*_) to model-based *UPP*_*ModEff*_ and decreases 94% (from 0.267 to 0.017) as we move from current practice to *UPP*_*FOA*_. These results are based on Logit demand and Generalized Leontief costs. A similar pattern is seen for many of the other scenarios as well, as documented in Table 3. Notably, in six of the eight scenarios, using *UPP*_*FOA*_ reduces total probability of false positive and false negatives to less than 0.02 (The exceptional cases are still the ones with log-linear demand as discussed above).

**Table 3:** Improvement in merger screening accuracy—5% tolerance threshold.

Generalized Leontief									
Logit Demand	NoEff	AvgEff	ModEff	FOA	Linear Demand	NoEff	AvgEff	ModEff	FOA
Type I error	0.579	0.175	0.022	0.010	Type I error	0.582	0.173	0.030	0.000
Type II error	0.035	0.092	0.035	0.008	Type II error	0.000	0.051	0.004	0.000
Total	0.614	0.267	0.057	0.017	Total	0.583	0.224	0.034	0.000
Absolute Gain w.r.t. AvgEff			0.210	0.250	Absolute Gain w.r.t. AvgEff			0.190	0.224
Relative Gain w.r.t. AvgEff (%)			78.78	93.53	Relative Gain w.r.t. AvgEff (%)			84.94	100.00
Log-Linear Demand	NoEff	AvgEff	ModEff	FOA	Almost Ideal Demand	NoEff	AvgEff	ModEff	FOA
Type I error	0.408	0.079	0.015	0.083	Type I error	0.430	0.084	0.014	0.003
Type II error	0.014	0.157	0.137	0.042	Type II error	0.008	0.131	0.091	0.010
Total	0.422	0.235	0.152	0.125	Total	0.438	0.215	0.105	0.013
Absolute Gain w.r.t. AvgEff			0.083	0.110	Absolute Gain w.r.t. AvgEff			0.110	0.202
Relative Gain w.r.t. AvgEff (%)			35.41	46.91	Relative Gain w.r.t. AvgEff (%)			51.30	93.90
Quadratic									
Logit Demand	NoEff	AvgEff	ModEff	FOA	Linear Demand	NoEff	AvgEff	ModEff	FOA
Type I error	0.334	0.172	0.075	0.006	Type I error	0.402	0.238	0.143	0.000
Type II error	0.000	0.019	0.000	0.001	Type II error	0.000	0.017	0.000	0.000
Total	0.334	0.190	0.075	0.007	Total	0.402	0.255	0.143	0.000
Absolute Gain w.r.t. AvgEff			0.115	0.184	Absolute Gain w.r.t. AvgEff			0.112	0.255
Relative Gain w.r.t. AvgEff (%)			60.61	96.53	Relative Gain w.r.t. AvgEff (%)			43.80	100.00
Log-Linear Demand	NoEff	AvgEff	ModEff	FOA	Almost Ideal Demand	NoEff	AvgEff	ModEff	FOA
Type I error	0.062	0.034	0.031	0.015	Type I error	0.122	0.041	0.031	0.003
Type II error	0.052	0.208	0.281	0.112	Type II error	0.010	0.114	0.176	0.003
Total	0.114	0.242	0.311	0.126	Total	0.132	0.155	0.206	0.007
Absolute Gain w.r.t. AvgEff			-0.069	0.116	Absolute Gain w.r.t. AvgEff			-0.051	0.148
Relative Gain w.r.t. AvgEff (%)			-28.48	47.87	Relative Gain w.r.t. AvgEff (%)			-33.12	95.75

The average reduction in total probability of making an error in these cases is 96%.

Moreover, in four scenarios, using *UPP*_*FOA*_ (over *UPP*_*AvgEff*_) reduces total probability of false positives and false negatives to less than 0.007. The average reduction in making a merger screening error in these four cases is 98%. In order to check robustness of these results, we ran the analysis with thresholds of 0 percent, 10 percent, and 15 percent as well. The results were similar.

As another robustness check, we use a different measure of the test’s accuracy, its F1 score. It is defined as the harmonic mean of the precision and recall ratios, which are defined as follows.

$$\begin{array}{c} \text{Precision}\;\text{Ratio}=\frac{\text{True}\;\text{test}\;\text{positives}}{\text{All}\;\text{test}\;\text{positives}\;} \end{array}$$

True test positives are those cases which are actually true and the test identifies them as true. All test positives are those cases which the test identifies as true (whether they are actually true or not is immaterial). The precision ratio measures truly predicted positives as a fraction of total predicted positives. In terms of Fig 4, mergers in the top right quadrant are true test positives and mergers to the right of the vertical line are all test positives.

$$\begin{array}{c} \text{Recall}\;\text{Ratio}=\frac{\text{True}\;\text{test}\;\text{positives}}{\text{All}\;\text{true}\;\text{positives}\;} \end{array}$$

True test positives are the same as before. All true positives are those cases that are actually true (whether the test identifies them as true or false is immaterial). The recall ratio measures truly predicted positives as a fraction of truly total positives. In terms of Fig 4, mergers in the top right quadrant are true test positives and mergers above the vertical line are all true positives.

F1 score is the harmonic mean of these ratios, given by

$$\begin{array}{c} \text{F1}\;\text{score}=[\frac{{\text{Precision}}^{-1}+{\text{Recall}}^{-1}}{2}{]}^{-1}=\frac{2*\text{Precision}*\text{Recall}}{\text{Precision}+\text{Recall}} \end{array}$$

Recall that for two positive numbers, the harmonic mean is (weakly) lower than the geometric mean, which is (weakly) lower than the arithmetic mean. Moreover, as both precision and recall ratios are in the unit interval, the F1 score is in the unit interval, and higher values for precision and recall ratios imply a higher F1 score.

Table 4 shows the precision ratio, recall ratio, and F1 score for each of the cases and computes improvement in F1 score, in a format analogous to Table 3.

**Table 4:** Improvement in merger screening accuracy—5% tolerance threshold.

Generalized Leontief									
Logit Demand	NoEff	AvgEff	ModEff	FOA	Linear Demand	NoEff	AvgEff	ModEff	FOA
Precision Ratio	0.243	0.423	0.894	0.955	Precision Ratio	0.238	0.431	0.857	1.000
Recall Ratio	0.840	0.582	0.842	0.965	Recall Ratio	0.998	0.718	0.978	1.000
F1 score	0.376	0.490	0.867	0.960	F1 score	0.385	0.539	0.914	1.000
Absolute Gain over AvgEff			0.285	0.470	Absolute Gain over AvgEff			0.375	0.461
Relative Gain over AvgEff (%)			76.96	95.89	Relative Gain over AvgEff (%)			69.51	85.56
Log-Linear Demand	NoEff	AvgEff	ModEff	FOA	Almost Ideal Demand	NoEff	AvgEff	ModEff	FOA
Precision Ratio	0.375	0.400	0.671	0.626	Precision Ratio	0.356	0.425	0.771	0.868
Recall Ratio	0.839	0.356	0.409	0.633	Recall Ratio	0.863	0.410	0.510	0.710
F1 score	0.518	0.377	0.508	0.629	F1 score	0.504	0.418	0.614	0.781
Absolute Gain over AvgEff			0.132	0.253	Absolute Gain over AvgEff			0.196	0.363
Relative Gain over AvgEff (%)			34.95	67.08	Relative Gain over AvgEff (%)			46.99	87.02
Quadratic									
Logit Demand	NoEff	AvgEff	ModEff	FOA	Linear Demand	NoEff	AvgEff	ModEff	FOA
Precision Ratio	0.563	0.706	0.852	0.986	Precision Ratio	0.474	0.592	0.717	1.000
Recall Ratio	1.000	0.957	1.000	0.999	Recall Ratio	0.999	0.952	0.999	1.000
F1 score	0.721	0.812	0.920	0.992	F1 score	0.643	0.730	0.835	1.000
Absolute Gain over AvgEff			0.108	0.180	Absolute Gain over AvgEff			0.104	0.270
Relative Gain over AvgEff (%)			13.23	22.14	Relative Gain over AvgEff (%)			14.31	36.92
Log-Linear Demand	NoEff	AvgEff	ModEff	FOA	Almost Ideal Demand	NoEff	AvgEff	ModEff	FOA
Precision Ratio	0.889	0.872	0.849	0.975	Precision Ratio	0.822	0.887	0.891	0.987
Recall Ratio	0.922	0.691	0.582	0.774	Recall Ratio	0.984	0.811	0.705	0.950
F1 score	0.906	0.771	0.690	0.863	F1 score	0.896	0.847	0.787	0.968
Absolute Gain over AvgEff			-0.081	0.092	Absolute Gain over AvgEff			-0.061	0.121
Relative Gain over AvgEff (%)			-10.46	11.93	Relative Gain over AvgEff (%)			-7.15	14.27

Table 4 shows a pattern similar to Table 3. As is well-known, *UPP*_*NoEff*_ is biased toward predicting positives, in the sense that if there is no adjustment for efficiencies, it predicts many mergers will raise prices even when prices may not truly increase, and therefore, adjustments for merger efficiencies are important both from a practitioner’s standpoint and from a theoretical standpoint. In Table 3, this shows up in a high incidence of Type I errors and low incidence of Type II errors for *UPP*_*NoEff*_. For precision and recall ratios, this implies that the precision ratio for *UPP*_*NoEff*_ would tend to be closer to zero and the recall ratio closer to one, as shown in Table 4.

Similar to the pattern in Table 3, F1 score increases 77% (from 0.490 to 0.867) as we move from current practice (using *UPP*_*AvgEff*_) to *UPP*_*ModEff*_, and it increases 96% (from 0.490 to 0.960) as we move from current practice to *UPP*_*FOA*_. These results are based on Logit demand and Generalized Leontief costs. A similar pattern is seen for many of the other scenarios as well.

In all eight scenarios, the F1 score with *UPP*_*FOA*_ is higher than that for the benchmark *UPP*_*AvgEff*_, with large gains in many cases. Notably, gains in F1 score are gains in harmonic mean, which weights lower numbers more, and therefore, makes it harder to get increases in the F1 score. Moreover, in five of the eight scenarios, the F1 score is very high, at a level 0.96 or above, and each of the precision and recall ratios in these cases are at 0.95 or above. Overall, these results support the results in Table 3. Additional details are presented in S1–S3 Tables.

Taken together, these results present more evidence of the benefit from including cost efficiencies in a manner guided by the model and the benefit of using a more accurate first-order approximation in UPP calculations. In particular, the results indicate that these UPP measures may be a good proxy for full merger simulations.

### Comparison to UPP with higher efficiency thresholds

We know that some adjustment to *UPP*_*NoEff*_ is needed to account for merger efficiencies. The previous analysis accounts for this by using the average efficiency generated by a given technology and showing how *UPP*_*ModEff*_ and *UPP*_*FOA*_ may improve upon that.

As another check on the validity of the results above, we consider different price increase thresholds for *UPP*_*NoEff*_ proposed in the literature and compare these to a stricter 0 percent threshold for *UPP*_*ModEff*_ and *UPP*_*FOA*_, as follows.

In the previous analysis, we consider a 5 percent threshold, due to its use as a benchmark for market definition in the hypothetical monopolist test, as described in 4.1.2 of HMG (2010) (Despite of the Agencies saying that the small but significant non transitory increase in price (SSNIP) is a threshold for market definition, as does not reflect their tolerance towards price increase, it is still a good indicator of what could potentially be considered anticompetitive.), as well as its proximity to the optimal threshold for UPP of four percent estimated in [25]. [1] suggest “using a starkly simple default value for efficiencies” that could, for example, be 10 percent. This would allow, in principle, to postpone more specific estimation of merger-specific efficiencies after evaluating the results of an initial screen, similar to suggestions in [32]. This 10 percent threshold is used by Miller et al. (2017) to analyze the occurrence of false positives and negatives in UPP. [44] analyzes antitrust cases evaluated by the FTC from 1993 until mid-2010 and concludes that an implicit benchmark used for UPP is 15 percent.

In order to provide a comparison to the current practice of using *UPP*_*NoEff*_ with higher thresholds, we compare probability of Type I and Type II errors using price increase thresholds of 5%, 10%, and 15% for *UPP*_*NoEff*_ and a stricter threshold of 0% for *UPP*_*ModEff*_ and *UPP*_*FOA*_. As shown in Table 5, in each of the eight scenarios, total probability of making type I and II errors with a 0% threshold for *UPP*_*ModEff*_ and *UPP*_*FOA*_ is lower than with a 15% threshold for *UPP*_*NoEff*_, and in many cases it is substantially lower.

**Table 5:** Baseline UPP with higher thresholds.

Generalized Leontief											
	Logit						Linear				
	*UPP*_*NoEff*_			*UPP*_*ModEff*_	*UPP*_*FOA*_		*UPP*_*NoEff*_			*UPP*_*ModEff*_	*UPP*_*FOA*_
	5%	10%	15%	0%	0%		5%	10%	15%	0%	0%
Type I error	0.579	0.436	0.295	0.000	0.020	Type I error	0.582	0.433	0.292	0.030	0.000
Type II error	0.035	0.026	0.021	0.060	0.001	Type II error	0.000	0.001	0.001	0.005	0.000
Total error	0.614	0.462	0.316	0.060	0.021	Total error	0.583	0.434	0.293	0.035	0.000
	Log-Linear						Almost Ideal				
	*UPP*_*NoEff*_			*UPP*_*ModEff*_	*UPP*_*FOA*_		*UPP*_*NoEff*_			*UPP*_*ModEff*_	*UPP*_*FOA*_
	5%	10%	15%	0%	0%		5%	10%	15%	0%	0%
Type I error	0.408	0.224	0.104	0.041	0.097	Type I error	0.430	0.244	0.119	0.038	0.005
Type II error	0.014	0.044	0.086	0.038	0.039	Type II error	0.008	0.024	0.050	0.022	0.010
Total error	0.422	0.267	0.190	0.079	0.136	Total error	0.438	0.268	0.169	0.059	0.015
Quadratic											
	Logit						Linear				
	*UPP*_*NoEff*_			*UPP*_*ModEff*_	*UPP*_*FOA*_		*UPP*_*NoEff*_			*UPP*_*ModEff*_	*UPP*_*FOA*_
	5%	10%	15%	0%	0%		5%	10%	15%	0%	0%
Type I error	0.334	0.406	0.306	0.000	0.000	Type I error	0.402	0.412	0.295	0.002	0.000
Type II error	0.000	0.000	0.000	0.000	0.000	Type II error	0.000	0.003	0.001	0.000	0.000
Total error	0.334	0.406	0.306	0.000	0.000	Total error	0.402	0.415	0.296	0.002	0.000
	Log-Linear						Almost Ideal				
	*UPP*_*NoEff*_			*UPP*_*ModEff*_	*UPP*_*FOA*_		*UPP*_*NoEff*_			*UPP*_*ModEff*_	*UPP*_*FOA*_
	5%	10%	15%	0%	0%		5%	10%	15%	0%	0%
Type I error	0.062	0.048	0.035	0.034	0.002	Type I error	0.122	0.096	0.063	0.031	0.001
Type II error	0.052	0.130	0.200	0.000	0.081	Type II error	0.010	0.037	0.074	0.000	0.003
Total error	0.114	0.178	0.235	0.034	0.083	Total error	0.132	0.133	0.137	0.032	0.004

Table shows the percentage of error types in merger screening for UPP baseline formulation (for 5, 10, and 15% tolerance threshold) when compared to UPP with model-based efficiencies and first-order approximation (with a strict tolerance of 0% threshold).

The difference between using higher thresholds for *UPP*_*NoEff*_ and a zero threshold for *UPP*_*ModEff*_ and *UPP*_*FOA*_ is starker when viewed through the lens of F1 score. As shown in Table 6, at higher thresholds for *UPP*_*NoEff*_, the F1 score actually goes down. Indeed, both the precision ratio and the recall ratio go down as well. In other words, when using *UPP*_*NoEff*_ at higher thresholds, true test positives as a fraction of total test positives go down and true test positives as a fraction of total true positives go down as well. Put differently, false test positives make up a growing share of all test positives and false test negatives make up a growing share of all true positives. In this sense, the test is increasingly likely to predict false positives and false negatives, and delivers decreasing F1 scores.

**Table 6:** Baseline UPP with higher thresholds.

Generalized Leontief											
	Logit						Linear				
	*UPP*_*NoEff*_			*UPP*_*ModEff*_	*UPP*_*FOA*_		*UPP*_*NoEff*_			*UPP*_*ModEff*_	*UPP*_*FOA*_
	5%	10%	15%	0%	0%		5%	10%	15%	0%	0%
Precision Ratio	0.243	0.180	0.147	0.999	0.953	Precision Ratio	0.238	0.186	0.157	0.915	1.000
Recall Ratio	0.840	0.787	0.711	0.855	0.997	Recall Ratio	0.998	0.992	0.982	0.984	1.000
F1 score	0.376	0.293	0.244	0.921	0.975	F1 score	0.385	0.313	0.270	0.949	1.000
	Log-Linear						Almost Ideal				
	*UPP*_*NoEff*_			*UPP*_*ModEff*_	*UPP*_*FOA*_		*UPP*_*NoEff*_			*UPP*_*ModEff*_	*UPP*_*FOA*_
	5%	10%	15%	0%	0%		5%	10%	15%	0%	0%
Precision Ratio	0.375	0.379	0.375	0.734	0.646	Precision Ratio	0.356	0.365	0.372	0.790	0.872
Recall Ratio	0.839	0.682	0.488	0.661	0.653	Recall Ratio	0.863	0.750	0.592	0.702	0.725
F1 score	0.518	0.487	0.424	0.695	0.650	F1 score	0.504	0.491	0.457	0.743	0.792
Quadratic											
	Logit						Linear				
	*UPP*_*NoEff*_			*UPP*_*ModEff*_	*UPP*_*FOA*_		*UPP*_*NoEff*_			*UPP*_*ModEff*_	*UPP*_*FOA*_
	5%	10%	15%	0%	0%		5%	10%	15%	0%	0%
Precision Ratio	0.563	0.238	0.118	1.000	1.000	Precision Ratio	0.474	0.225	0.150	0.998	1.000
Recall Ratio	1.000	1.000	1.000	1.000	1.000	Recall Ratio	0.999	0.979	0.975	1.000	1.000
F1 score	0.721	0.384	0.211	1.000	1.000	F1 score	0.643	0.366	0.260	0.999	1.000
	Log-Linear						Almost Ideal				
	*UPP*_*NoEff*_			*UPP*_*ModEff*_	*UPP*_*FOA*_		*UPP*_*NoEff*_			*UPP*_*ModEff*_	*UPP*_*FOA*_
	5%	10%	15%	0%	0%		5%	10%	15%	0%	0%
Precision Ratio	0.889	0.825	0.719	0.966	0.997	Precision Ratio	0.822	0.767	0.711	0.968	0.998
Recall Ratio	0.922	0.755	0.532	1.000	0.836	Recall Ratio	0.984	0.912	0.759	0.999	0.952
F1 score	0.906	0.788	0.612	0.983	0.909	F1 score	0.896	0.833	0.734	0.984	0.974

Table shows the F1 scores and its components in merger screening for UPP baseline formulation (for 5, 10, and 15% tolerance threshold) when compared to UPP with model-based efficiencies and first-order approximation (with a strict tolerance of 0% threshold).

In contrast, the F1 scores remain high for both *UPP*_*ModEff*_ and *UPP*_*FOA*_ at a zero percent threshold.

## Curvature of merger-specific efficiencies

The analysis above focuses on the case when cost complementarities across merging firms may be proxied by Generalized Leontief or Quadratic costs. In this section, we present additional results for the case when merger-specific efficiencies may have different curvature, as follows. Consider merger-specific efficiencies of the form:

$$\begin{array}{c} \phi \left(Q_{i},Q_{j}\right)=\alpha_{ij}Q_{i}^{\gamma }Q_{j}^{\gamma } \end{array}$$

where *γ* ∈ [0, 1] parameterizes curvature of *ϕ*. The case $\gamma =\frac{1}{2}$ corresponds to the case of Generalized Leontief costs (and *γ* = 1 is used for efficiencies in the case of Quadratic costs). The restriction *γ* ∈ [0, 1] implies that $-\alpha_{ij}Q_{i}^{\gamma }Q_{j}^{\gamma }$ is a convex function, and therefore, yields a well-defined concave profit-maximization problem. This provides a tractable class of merger-specific efficiencies with different curvature.

In order to keep the analysis manageable, consider the case of Generalized Leontief costs with flexible merger-specific efficiencies, formalized as follows.

$$\begin{array}{c} C\left(Q_{i},Q_{j}\right)=\alpha_{ii}Q_{i}+\alpha_{jj}Q_{j}-\alpha_{ij}Q_{i}^{\gamma }Q_{j}^{\gamma } \end{array}$$

The Monte Carlo simulation is conducted as earlier with four demand systems, with this cost function, and using an additional random draw for the parameter *γ* ∈ [0, 1]. This yields another 20,000 mergers (4 scenarios, each with 5,000 mergers). Results for these scenarios are summarized in Tables 7, 8, and 9.

**Table 7:** Improvement in price prediction.

Curvature									
Logit Demand	NoEff	AvgEff	ModEff	FOA	Linear Demand	NoEff	AvgEff	ModEff	FOA
	APE (p.p.)					APE (p.p.)			
Median	10.71	9.00	0.90	0.08	Median	10.73	8.34	1.24	0.00
Absolute Gain over AvgEff			8.10	8.93	Absolute Gain over AvgEff			7.09	8.34
Relative Gain over AvgEff (%)			90.00	99.16	Relative Gain over AvgEff (%)			85.11	100.00
	RPE (%)					RPE (%)			
Median	10.09	8.67	0.86	0.07	Median	10.51	8.16	1.20	0.00
Absolute Gain over AvgEff			7.81	8.60	Absolute Gain over AvgEff			6.95	8.16
Relative Gain over AvgEff (%)			90.12	99.19	Relative Gain over AvgEff (%)			85.25	100.00
Log-Linear Demand	NoEff	AvgEff	ModEff	FOA	Almost Ideal Demand	NoEff	AvgEff	ModEff	FOA
	APE (p.p.)					APE (p.p.)			
Median	13.16	15.87	9.32	6.29	Median	8.52	11.97	3.61	0.35
Absolute Gain over AvgEff			6.55	9.58	Absolute Gain over AvgEff			8.35	11.62
Relative Gain over AvgEff (%)			41.27	60.38	Relative Gain over AvgEff (%)			69.81	97.05
	RPE (%)					RPE (%)			
Median	12.33	15.03	8.98	5.77	Median	8.10	11.45	3.58	0.34
Absolute Gain over AvgEff			6.05	9.27	Absolute Gain over AvgEff			7.87	11.11
Relative Gain over AvgEff (%)			40.27	61.63	Relative Gain over AvgEff (%)			68.75	97.05

**Table 8:** Improvement in merger screening accuracy—5% tolerance threshold.

Curvature—Total Errors									
Logit Demand	NoEff	AvgEff	ModEff	FOA	Linear Demand	NoEff	AvgEff	ModEff	FOA
Type I error	0.577	0.190	0.042	0.005	Type I error	0.579	0.186	0.047	0.000
Type II error	0.020	0.089	0.019	0.005	Type II error	0.000	0.064	0.002	0.000
Total	0.596	0.279	0.061	0.010	Total	0.580	0.250	0.049	0.000
Absolute Gain over AvgEff			0.217	0.268	Absolute Gain over AvgEff			0.201	0.250
Relative Gain over AvgEff (%)			78.00	96.31	Relative Gain over AvgEff (%)			80.23	100.00
Log-Linear Demand	NoEff	AvgEff	ModEff	FOA	Almost Ideal Demand	NoEff	AvgEff	ModEff	FOA
Type I error	0.325	0.085	0.021	0.075	Type I error	0.369	0.086	0.018	0.006
Type II error	0.021	0.237	0.216	0.062	Type II error	0.003	0.175	0.131	0.011
Total	0.346	0.322	0.237	0.137	Total	0.372	0.261	0.150	0.017
Absolute Gain over AvgEff			0.085	0.186	Absolute Gain over AvgEff			0.111	0.244
Relative Gain over AvgEff (%)			26.38	57.60	Relative Gain over AvgEff (%)			42.68	93.58
Curvature—F1 score									
Logit Demand	NoEff	AvgEff	ModEff	FOA	Linear Demand	NoEff	AvgEff	ModEff	FOA
Precision Ratio	0.246	0.384	0.817	0.972	Precision Ratio	0.242	0.395	0.795	1.000
Recall Ratio	0.904	0.570	0.906	0.975	Recall Ratio	0.998	0.656	0.987	1.000
F1 score	0.386	0.459	0.859	0.974	F1 score	0.390	0.493	0.881	1.000
Absolute Gain over AvgEff			0.400	0.515	Absolute Gain over AvgEff			0.388	0.507
Relative Gain over AvgEff (%)			87.23	112.09	Relative Gain over AvgEff (%)			78.73	102.87
Log-Linear Demand	NoEff	AvgEff	ModEff	FOA	Almost Ideal Demand	NoEff	AvgEff	ModEff	FOA
Precision Ratio	0.511	0.501	0.766	0.748	Precision Ratio	0.458	0.518	0.853	0.890
Recall Ratio	0.871	0.344	0.393	0.685	Recall Ratio	0.916	0.417	0.513	0.789
F1 score	0.644	0.408	0.520	0.715	F1 score	0.611	0.462	0.641	0.836
Absolute Gain over AvgEff			0.112	0.308	Absolute Gain over AvgEff			0.179	0.374
Relative Gain over AvgEff (%)			27.56	75.54	Relative Gain over AvgEff (%)			38.67	80.92

**Table 9:** Baseline UPP with higher thresholds.

Curvature—Total Errors											
	Logit						Linear				
	*UPP*_*NoEff*_			*UPP*_*ModEff*_	*UPP*_*FOA*_		*UPP*_*NoEff*_			*UPP*_*ModEff*_	*UPP*_*FOA*_
	5%	10%	15%	0%	0%		5%	10%	15%	0%	0%
Type I error	0.577	0.467	0.322	0.001	0.011	Type I error	0.579	0.457	0.308	0.041	0.000
Type II error	0.020	0.015	0.011	0.036	0.002	Type II error	0.000	0.002	0.001	0.009	0.000
Total error	0.596	0.482	0.333	0.037	0.013	Total error	0.580	0.459	0.309	0.050	0.000
	Log-Linear						Almost Ideal				
	*UPP*_*NoEff*_			*UPP*_*ModEff*_	*UPP*_*FOA*_		*UPP*_*NoEff*_			*UPP*_*ModEff*_	*UPP*_*FOA*_
	5%	10%	15%	0%	0%		5%	10%	15%	0%	0%
Type I error	0.325	0.205	0.117	0.057	0.081	Type I error	0.369	0.248	0.136	0.052	0.008
Type II error	0.021	0.059	0.089	0.054	0.059	Type II error	0.003	0.017	0.031	0.037	0.011
Total error	0.346	0.265	0.206	0.111	0.141	Total error	0.372	0.265	0.168	0.089	0.019
Curvature—F1 score											
	Logit						Linear				
	*UPP*_*NoEff*_			*UPP*_*ModEff*_	*UPP*_*FOA*_		*UPP*_*NoEff*_			*UPP*_*ModEff*_	*UPP*_*FOA*_
	5%	10%	15%	0%	0%		5%	10%	15%	0%	0%
Precision Ratio	0.246	0.122	0.072	0.998	0.979	Precision Ratio	0.242	0.142	0.111	0.920	1.000
Recall Ratio	0.904	0.814	0.689	0.933	0.996	Recall Ratio	0.998	0.970	0.975	0.981	1.000
F1 score	0.386	0.212	0.131	0.964	0.987	F1 score	0.390	0.247	0.200	0.949	1.000
	Log-Linear						Almost Ideal				
	*UPP*_*NoEff*_			*UPP*_*ModEff*_	*UPP*_*FOA*_		*UPP*_*NoEff*_			*UPP*_*ModEff*_	*UPP*_*FOA*_
	5%	10%	15%	0%	0%		5%	10%	15%	0%	0%
Precision Ratio	0.511	0.475	0.443	0.832	0.799	Precision Ratio	0.458	0.406	0.404	0.863	0.929
Recall Ratio	0.871	0.712	0.546	0.730	0.716	Recall Ratio	0.916	0.821	0.707	0.751	0.804
F1 score	0.644	0.570	0.489	0.777	0.755	F1 score	0.611	0.543	0.514	0.803	0.862

Table shows the F1 scores and its components, as well as total errors, in merger screening for UPP baseline formulation (for 5, 10, and 15% tolerance threshold) when compared to UPP with model-based efficiencies and first-order approximation (with a strict tolerance of 0% threshold).

As shown in Table 7, there are substantial gains in price prediction accuracy (over *UPP*_*AvgEff*_) at the median similar to the corresponding cases in Table 2. As shown in the top panel in Table 8, there are notable reductions in total errors, and as shown in the bottom panel in Table 8, there are substantial improvements in F1 score as well.

As shown in Table 9, *UPP*_*NoEff*_ with higher thresholds continues to perform worse in terms of total errors, as compared to *UPP*_*ModEff*_ and *UPP*_*FOA*_ at a zero threshold. Moreover, *UPP*_*NoEff*_ with higher thresholds continues to perform poorly in terms of F1 score, whereas *UPP*_*ModEff*_ and *UPP*_*FOA*_ at zero threshold continue to possess substantially higher F1 scores. Additional details are presented in S4 and S5 Tables.

Overall, these results support the previous analysis. We also performed the analysis for separate values of *γ*. We used $\gamma =\frac{1}{n}$ for *n* = 1, …, 30 yielding 360,000 mergers (4 demand systems and 30 cost systems, for a total of 120 scenarios, each with 3,000 mergers). The results are similar.

## Conclusion

We investigate the accuracy of UPP as a tool in antitrust analysis by extending the standard UPP formulation to include merger-specific cost efficiencies. We include cost efficiencies in a tractable manner in the existing theoretical framework and derive the related UPP formulations.

The efficacy of the new UPP formulations is analyzed using Monte Carlo simulation for 8 different scenarios; four demand systems (Logit, Linear, Log-Linear, and Almost ideal) and two merger-specific, cost complementarity systems (Generalized Leontief and Quadratic). For each scenario we simulate 5,000 mergers, for a total of 40,000 mergers.

We find that the new UPP formulations yield substantial gains in post-merger price prediction and in merger screening accuracy. The results are robust to several additional analyses, including using F1 score and allowing for more flexible merger-specific efficiencies. The results show that including cost efficiencies in a manner guided by the theoretical model may yield substantial improvements in accuracy of UPP as a tool in antitrust analysis.

## Supporting information

S1 Fig Accuracy of prediction—Linear demand, Generalized Leontief cost. First row shows the distribution of the true post merger prices against the predicted post merger prices using different UPP calculations and own goods’ efficiencies included in the computation. Second row shows the same for both goods’ efficiencies.
(TIF)

S2 Fig Absolute prediction errors—Linear demand, Generalized Leontief cost. Portrays density kernels for absolute prediction errors, as well as the median absolute prediction error, first and third quartile for each specification.
(TIF)

S3 Fig Relative prediction errors—Linear demand, Generalized Leontief cost. Portrays density kernels for relative prediction errors, as well as the median relative prediction error, first and third quartile for each specification.
(TIF)

S4 Fig Accuracy of prediction—Log-linear demand, Generalized Leontief cost. First row shows the distribution of the true post merger prices against the predicted post merger prices using different UPP calculations and own goods’ efficiencies included in the computation. Second row shows the same for both goods’ efficiencies.
(TIF)

S5 Fig Absolute prediction errors—Log-linear demand, Generalized Leontief cost. Portrays density kernels for absolute prediction errors, as well as the median absolute prediction error, first and third quartile for each specification.
(TIF)

S6 Fig Relative prediction errors—Log-linear demand, Generalized Leontief cost. Portrays density kernels for relative prediction errors, as well as the median relative prediction error, first and third quartile for each specification.
(TIF)

S7 Fig Accuracy of prediction—Almost ideal demand, Generalized Leontief cost. First row shows the distribution of the true post merger prices against the predicted post merger prices using different UPP calculations and own goods’ efficiencies included in the computation. Second row shows the same for both goods’ efficiencies.
(TIF)

S8 Fig Absolute prediction errors—Almost ideal demand, Generalized Leontief cost. Portrays density kernels for absolute prediction errors, as well as the median absolute prediction error, first and third quartile for each specification.
(TIF)

S9 Fig Relative prediction errors—Almost ideal demand, Generalized Leontief cost. Portrays density kernels for relative prediction errors, as well as the median relative prediction error, first and third quartile for each specification.
(TIF)

S10 Fig Accuracy of prediction—Logit demand, quadratic cost. First row shows the distribution of the true post merger prices against the predicted post merger prices using different UPP calculations and own goods’ efficiencies included in the computation. Second row shows the same for both goods’ efficiencies.
(TIF)

S11 Fig Absolute prediction errors—Logit demand, quadratic cost. Portrays density kernels for absolute prediction errors, as well as the median absolute prediction error, first and third quartile for each specification.
(TIF)

S12 Fig Relative prediction errors—Logit demand, quadratic cost. Portrays density kernels for relative prediction errors, as well as the median relative prediction error, first and third quartile for each specification.
(TIF)

S13 Fig Accuracy of prediction—Linear demand, quadratic cost. First row shows the distribution of the true post merger prices against the predicted post merger prices using different UPP calculations and own goods’ efficiencies included in the computation. Second row shows the same for both goods’ efficiencies.
(TIF)

S14 Fig Absolute prediction errors—Linear demand, quadratic cost. Portrays density kernels for absolute prediction errors, as well as the median absolute prediction error, first and third quartile for each specification.
(TIF)

S15 Fig Relative prediction errors—Linear demand, quadratic cost. Portrays density kernels for relative prediction errors, as well as the median relative prediction error, first and third quartile for each specification.
(TIF)

S16 Fig Accuracy of prediction—Log-linear demand, quadratic cost. First row shows the distribution of the true post merger prices against the predicted post merger prices using different UPP calculations and own goods’ efficiencies included in the computation. Second row shows the same for both goods’ efficiencies.
(TIF)

S17 Fig Absolute prediction errors—Log-linear demand, quadratic cost. Portrays density kernels for absolute prediction errors, as well as the median absolute prediction error, first and third quartile for each specification.
(TIF)

S18 Fig Relative prediction errors—Log-linear demand, quadratic cost. Portrays density kernels for relative prediction errors, as well as the median relative prediction error, first and third quartile for each specification.
(TIF)

S19 Fig Accuracy of prediction—Almost ideal demand, quadratic cost. First row shows the distribution of the true post merger prices against the predicted post merger prices using different UPP calculations and own goods’ efficiencies included in the computation. Second row shows the same for both goods’ efficiencies.
(TIF)

S20 Fig Absolute prediction errors—Almost ideal demand, quadratic cost. Portrays density kernels for absolute prediction errors, as well as the median absolute prediction error, first and third quartile for each specification.
(TIF)

S21 Fig Relative prediction errors—Almost ideal demand, quadratic cost. Portrays density kernels for relative prediction errors, as well as the median relative prediction error, first and third quartile for each specification.
(TIF)

S1 Dataset Monte Carlo dataset. (CSV)

S1 Table Improvement in merger screening accuracy—10% tolerance threshold. (PDF)

S2 Table Improvement in merger screening accuracy—0% tolerance threshold. (PDF)

S3 Table Improvement in merger screening accuracy—10% tolerance threshold. (PDF)

S4 Table Improvement in merger screening accuracy—0% tolerance threshold. (PDF)

S5 Table Improvement in merger screening accuracy—10% tolerance threshold. (PDF)
