# Supplementary figures and images for: Antitrust analysis with upward pricing pressure and cost efficiencies
Source: PLoS One. 2020 Jan 8;15(1):e0227418. doi: 10.1371/journal.pone.0227418 (PMC6949007; doi:10.1371/journal.pone.0227418)

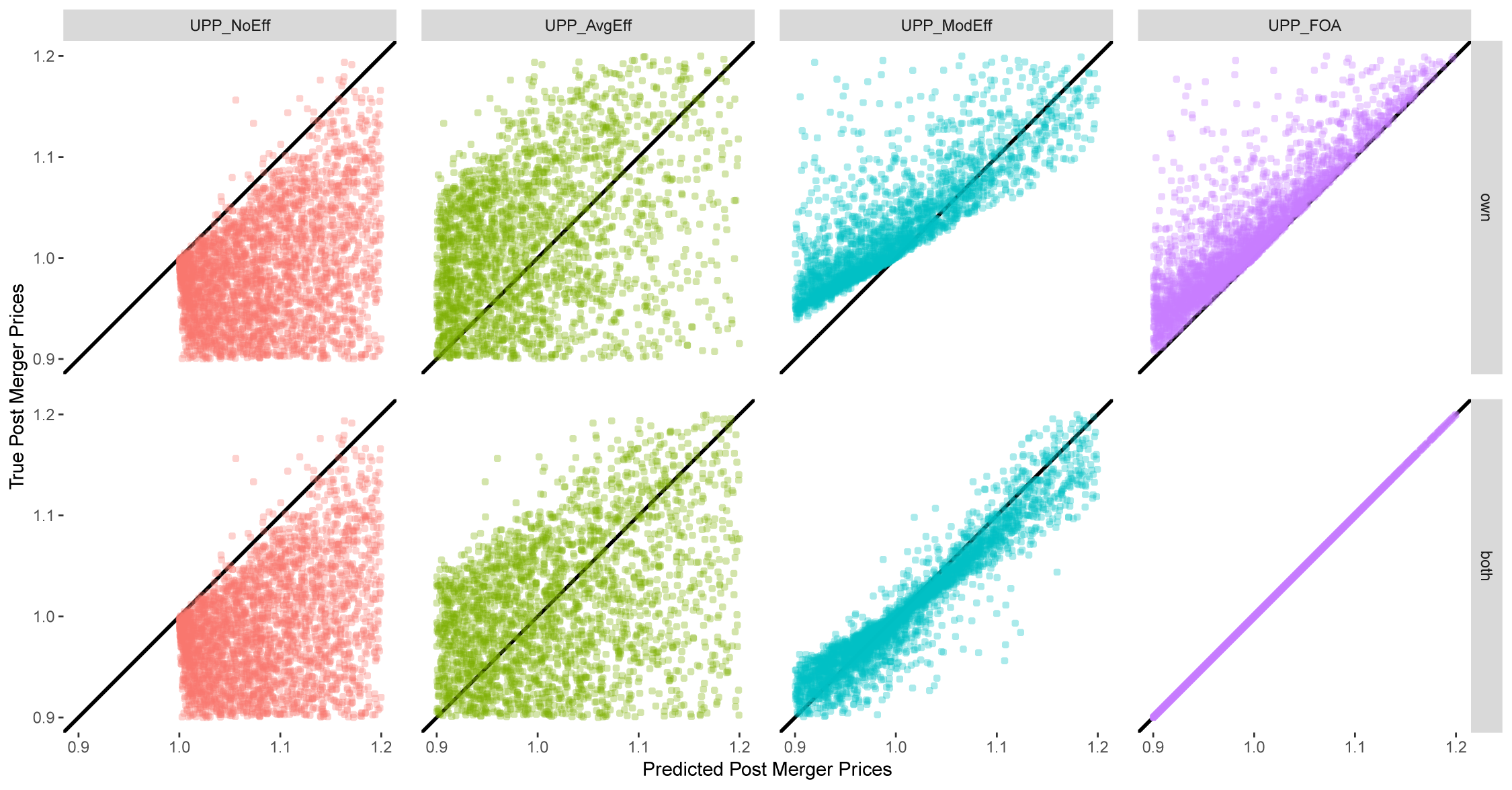

Supplement: S1 Fig — First row shows the distribution of the true post merger prices against the predicted post merger prices using different UPP calculations and own goods’ efficiencies included in the computation. Second row shows the same for both goods’ efficiencies. (TIF) [file pone.0227418.s001.tif]

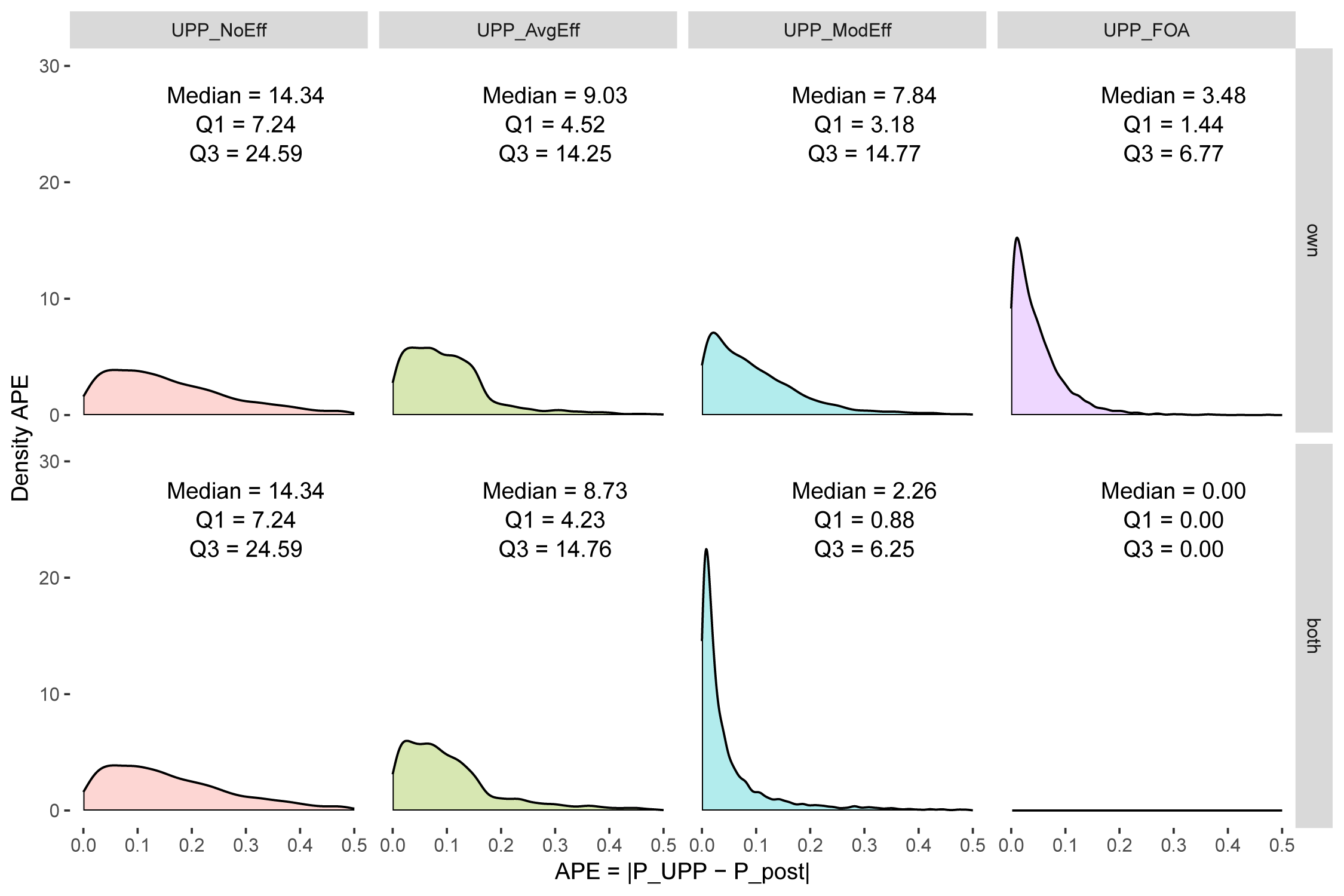

Supplement: S2 Fig — Portrays density kernels for absolute prediction errors, as well as the median absolute prediction error, first and third quartile for each specification. (TIF) [file pone.0227418.s002.tif]

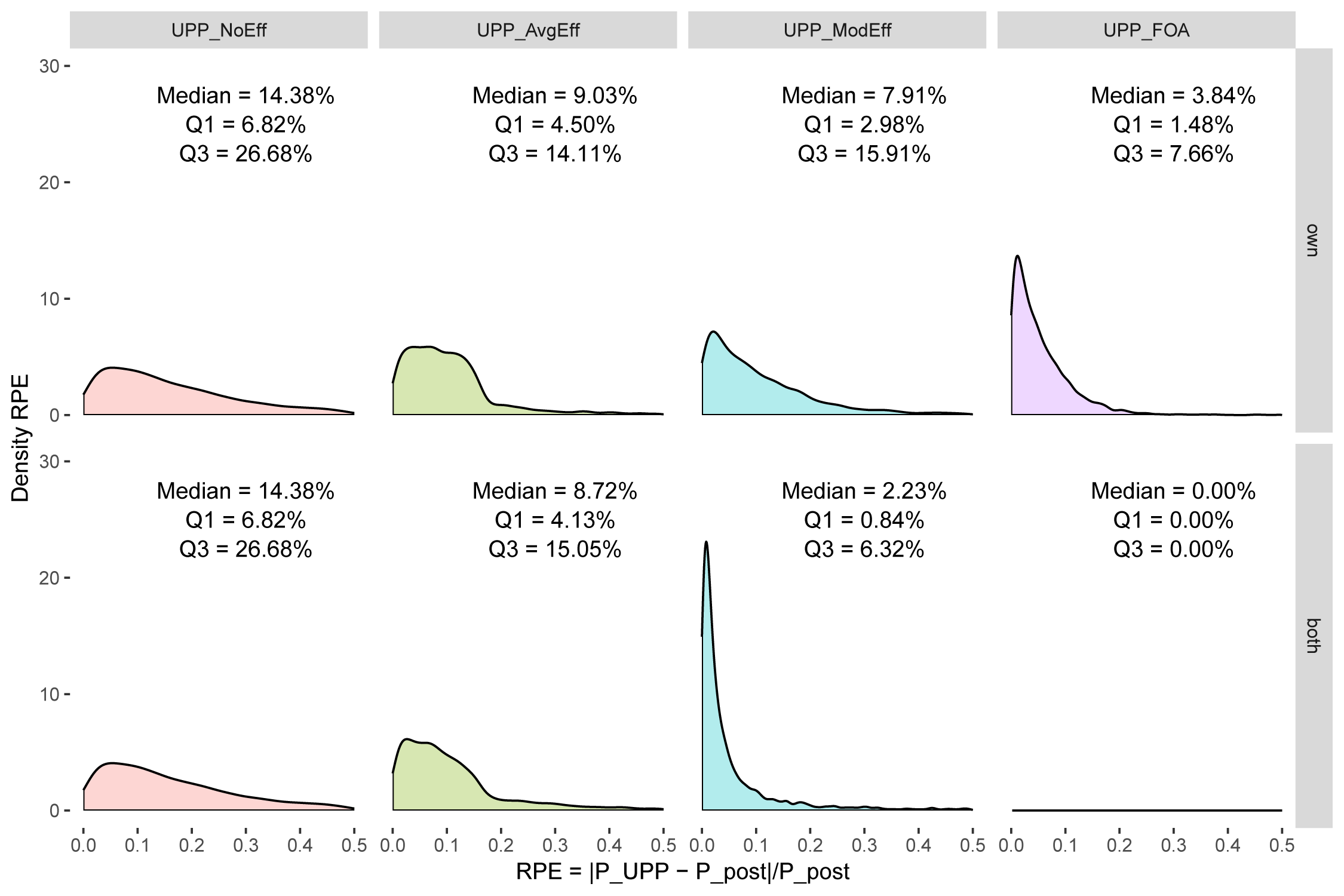

Supplement: S3 Fig — Portrays density kernels for relative prediction errors, as well as the median relative prediction error, first and third quartile for each specification. (TIF) [file pone.0227418.s003.tif]

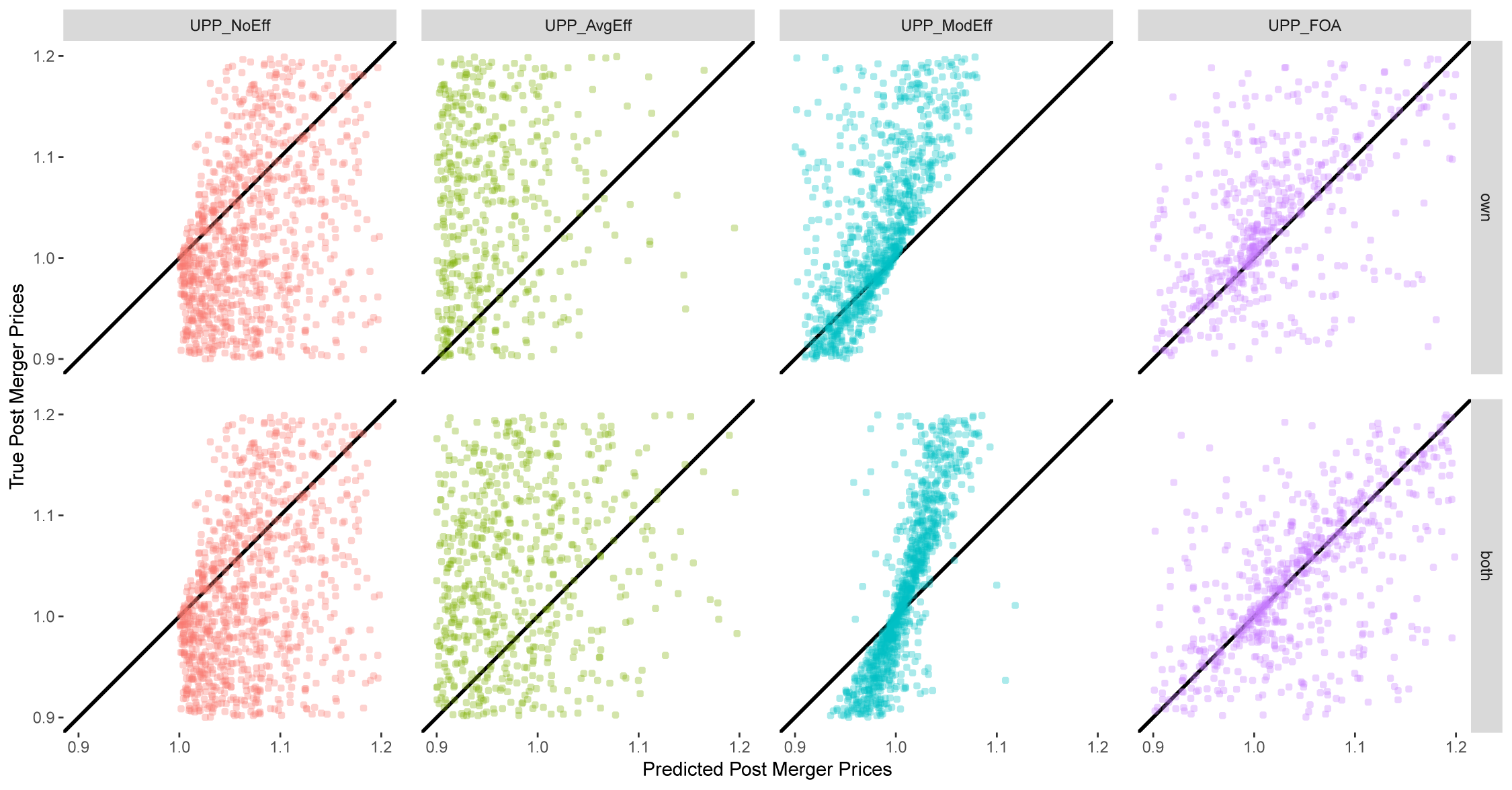

Supplement: S4 Fig — First row shows the distribution of the true post merger prices against the predicted post merger prices using different UPP calculations and own goods’ efficiencies included in the computation. Second row shows the same for both goods’ efficiencies. (TIF) [file pone.0227418.s004.tif]

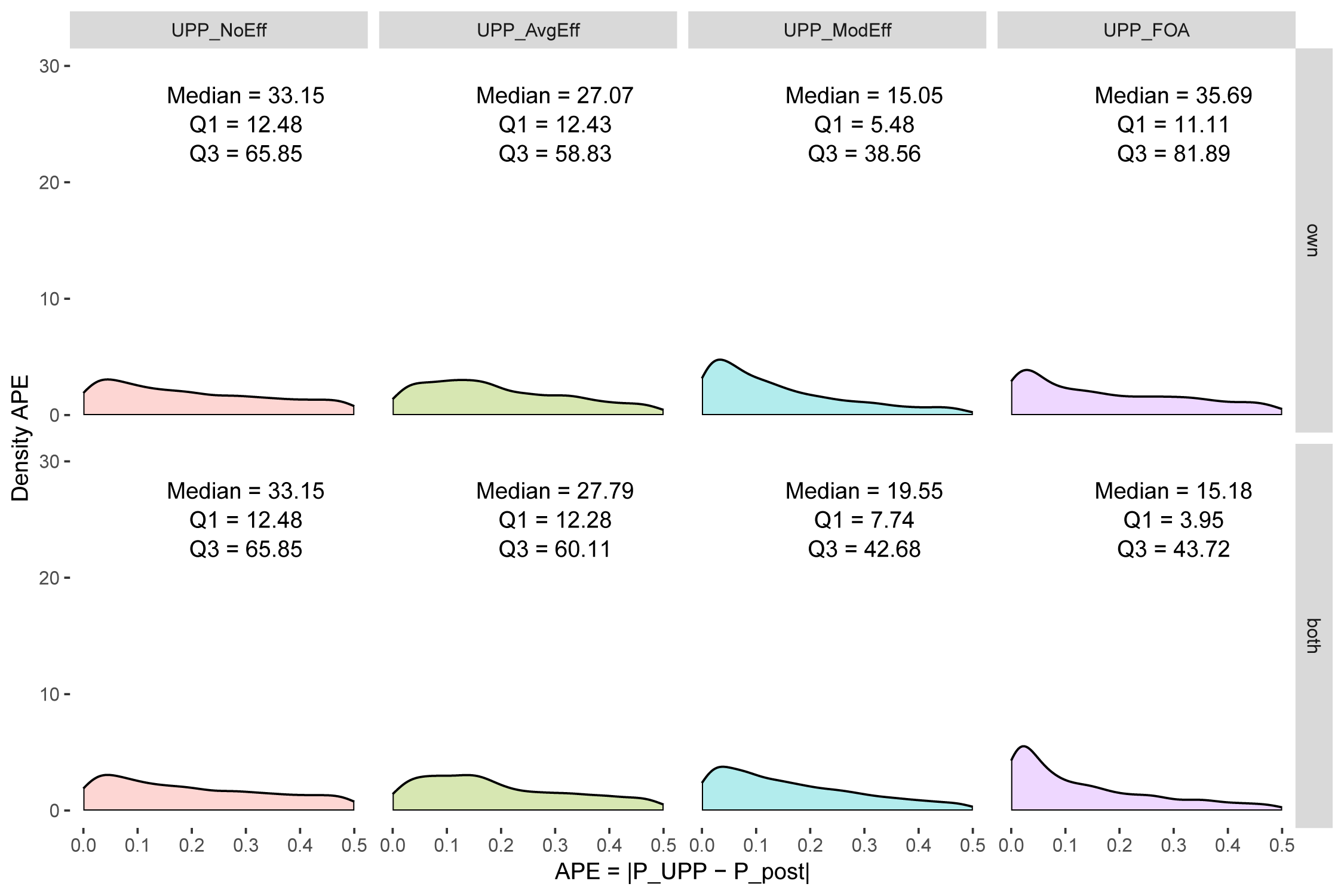

Supplement: S5 Fig — Portrays density kernels for absolute prediction errors, as well as the median absolute prediction error, first and third quartile for each specification. (TIF) [file pone.0227418.s005.tif]

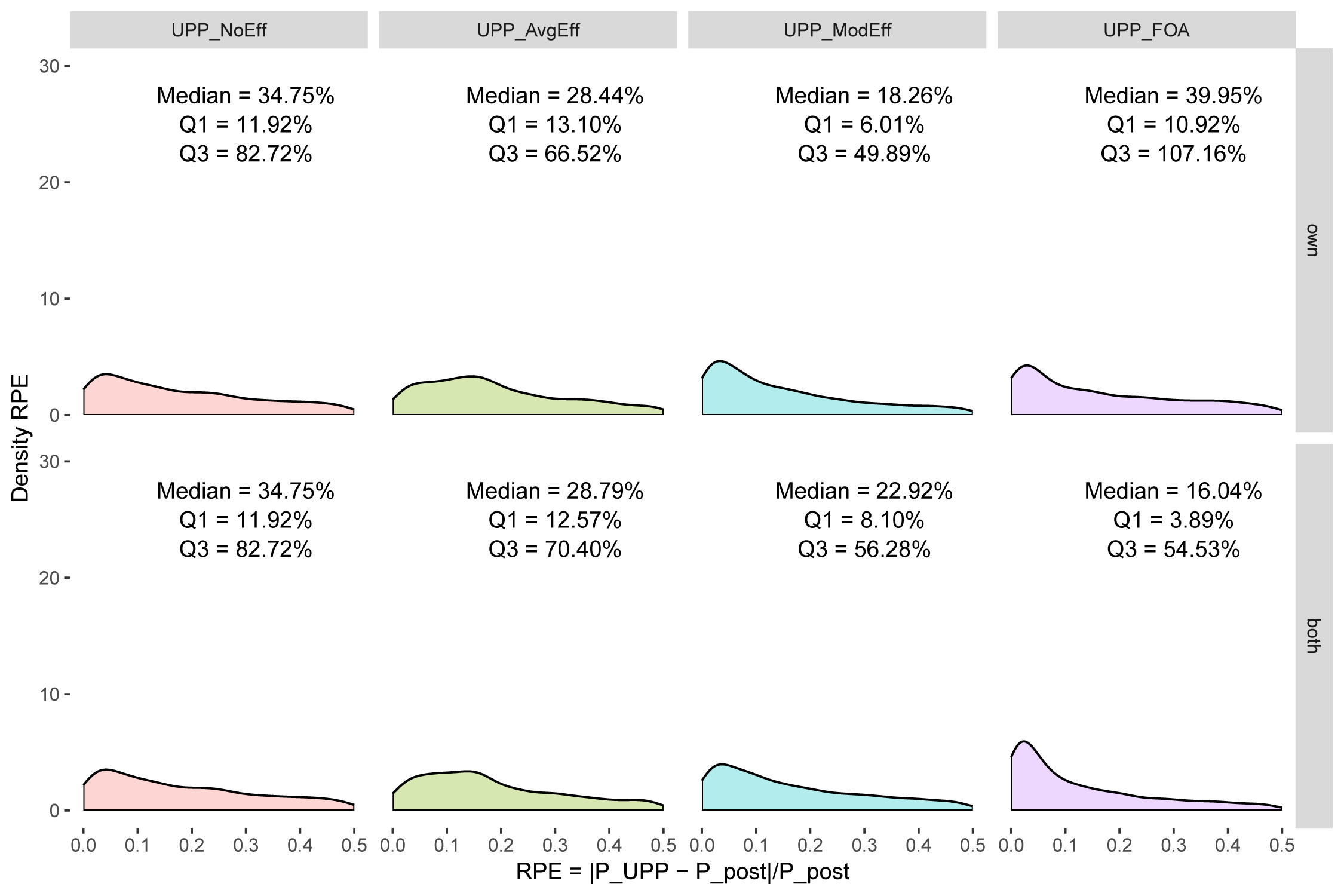

Supplement: S6 Fig — Portrays density kernels for relative prediction errors, as well as the median relative prediction error, first and third quartile for each specification. (TIF) [file pone.0227418.s006.tif]

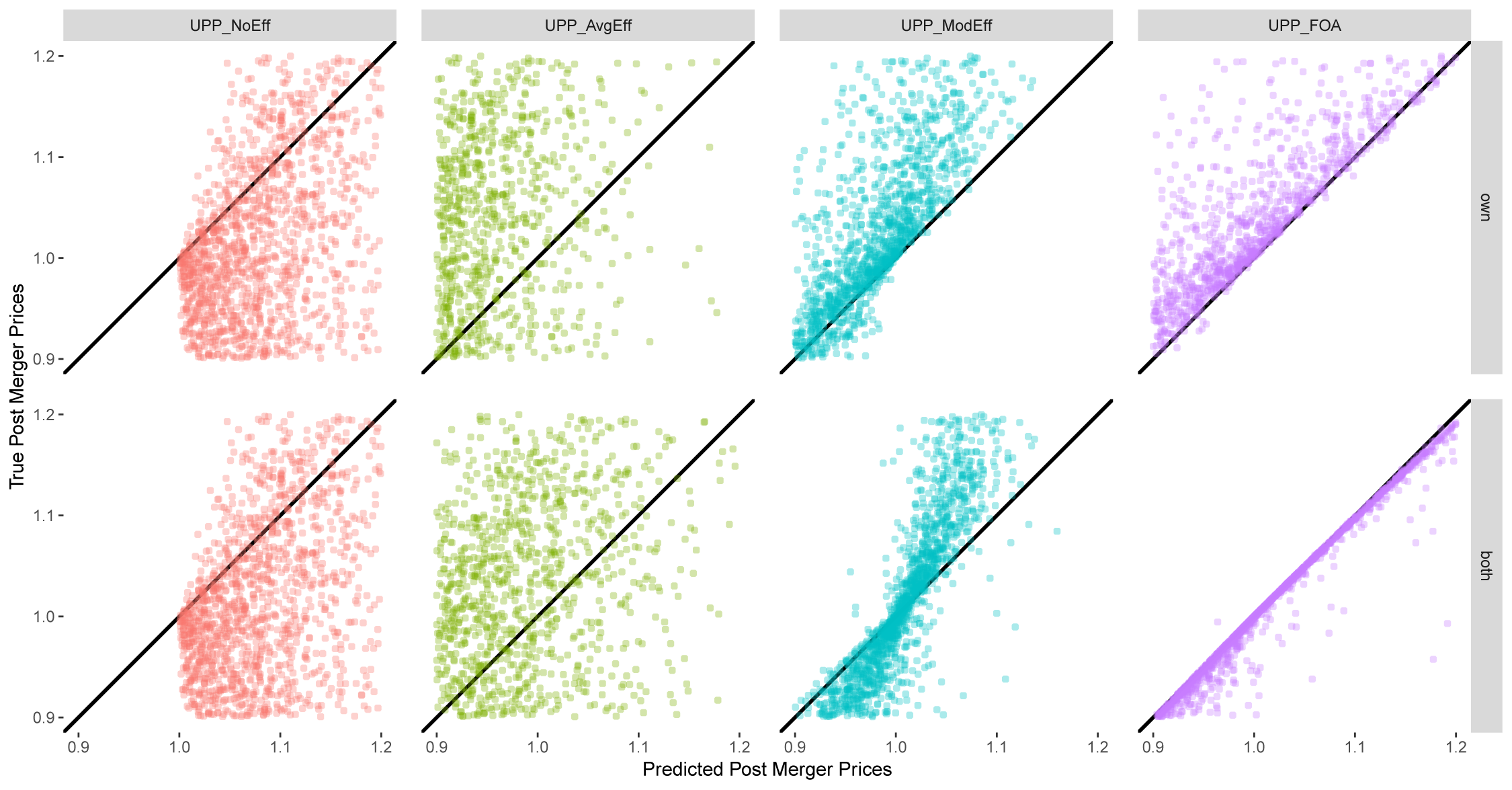

Supplement: S7 Fig — First row shows the distribution of the true post merger prices against the predicted post merger prices using different UPP calculations and own goods’ efficiencies included in the computation. Second row shows the same for both goods’ efficiencies. (TIF) [file pone.0227418.s007.tif]

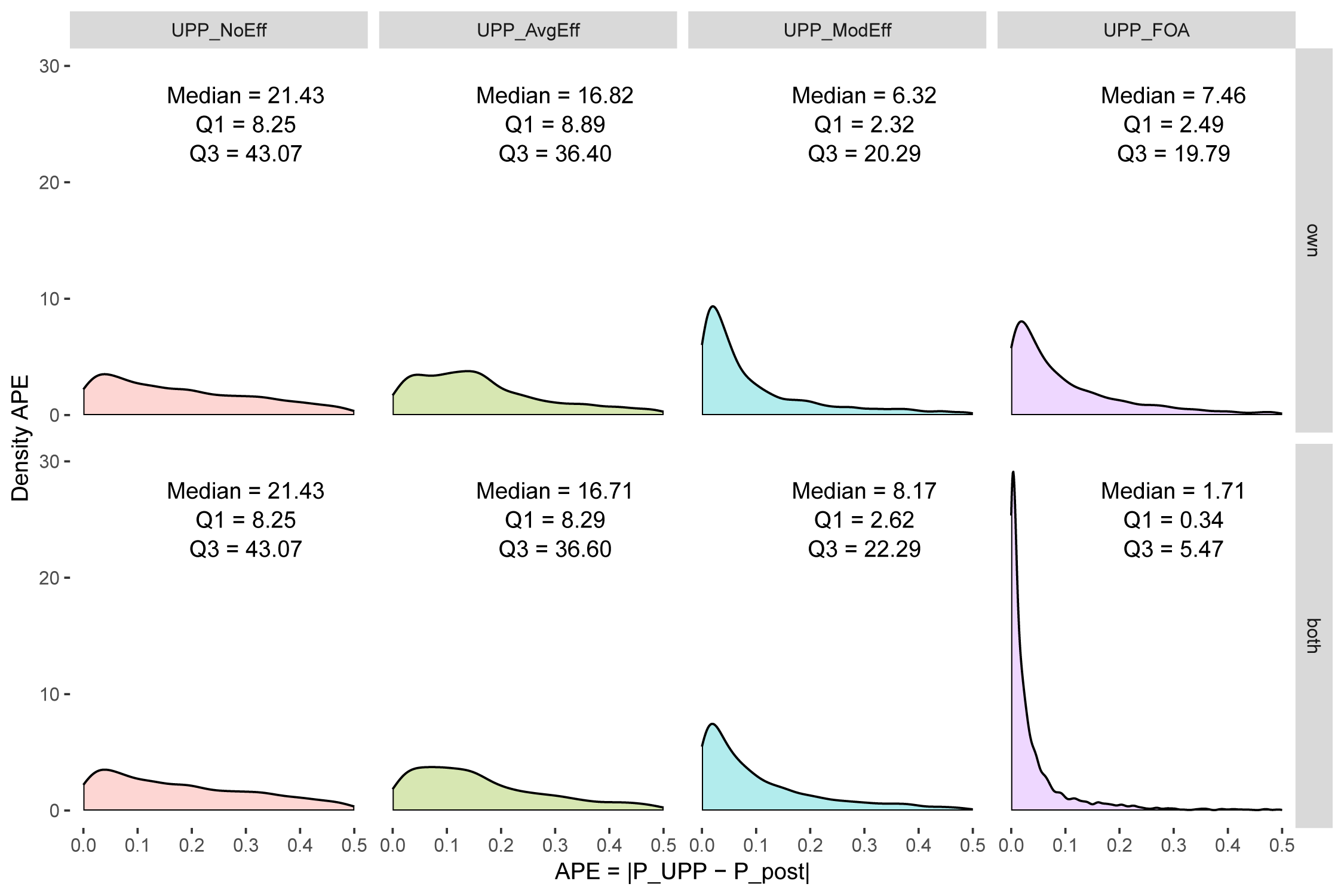

Supplement: S8 Fig — Portrays density kernels for absolute prediction errors, as well as the median absolute prediction error, first and third quartile for each specification. (TIF) [file pone.0227418.s008.tif]

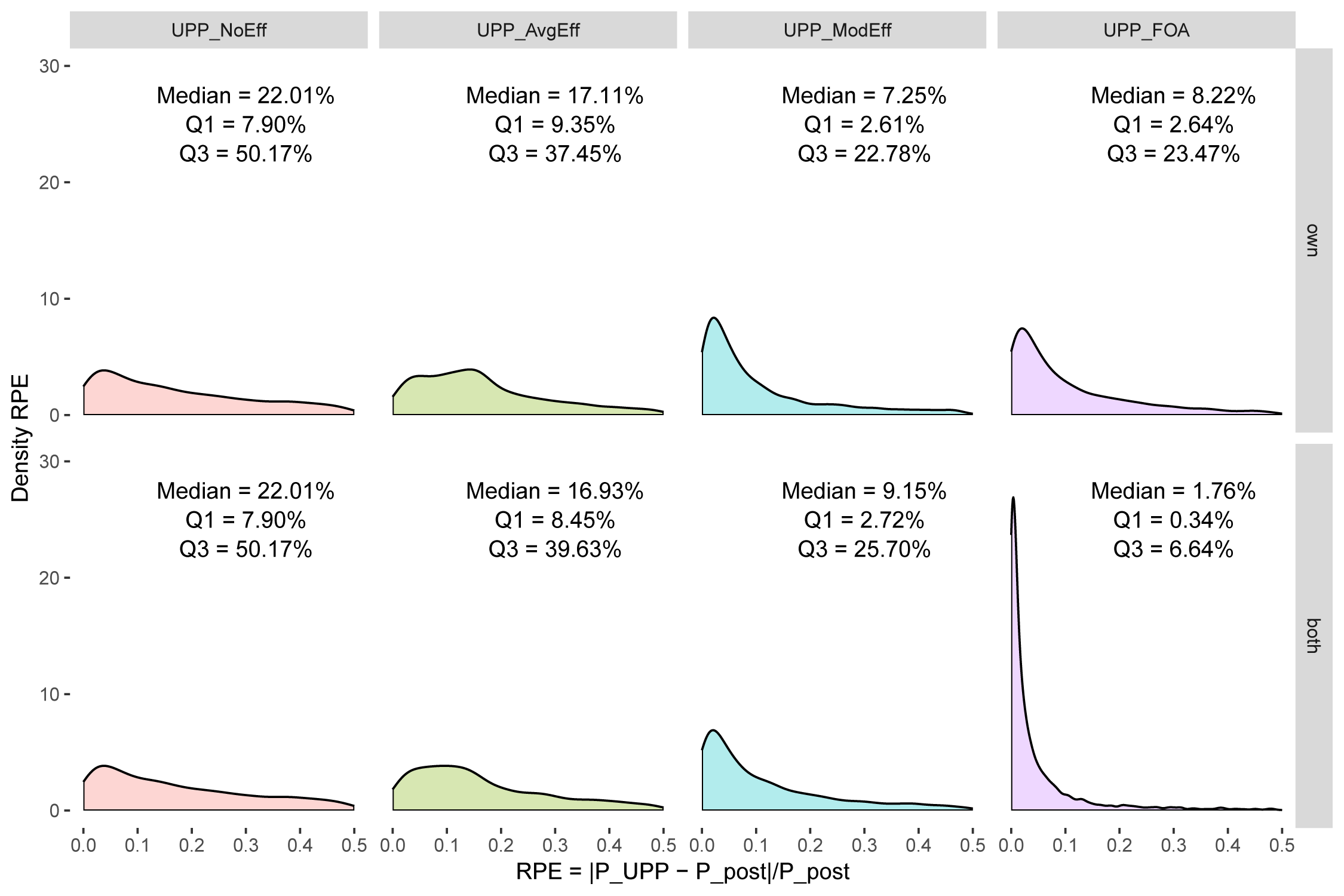

Supplement: S9 Fig — Portrays density kernels for relative prediction errors, as well as the median relative prediction error, first and third quartile for each specification. (TIF) [file pone.0227418.s009.tif]

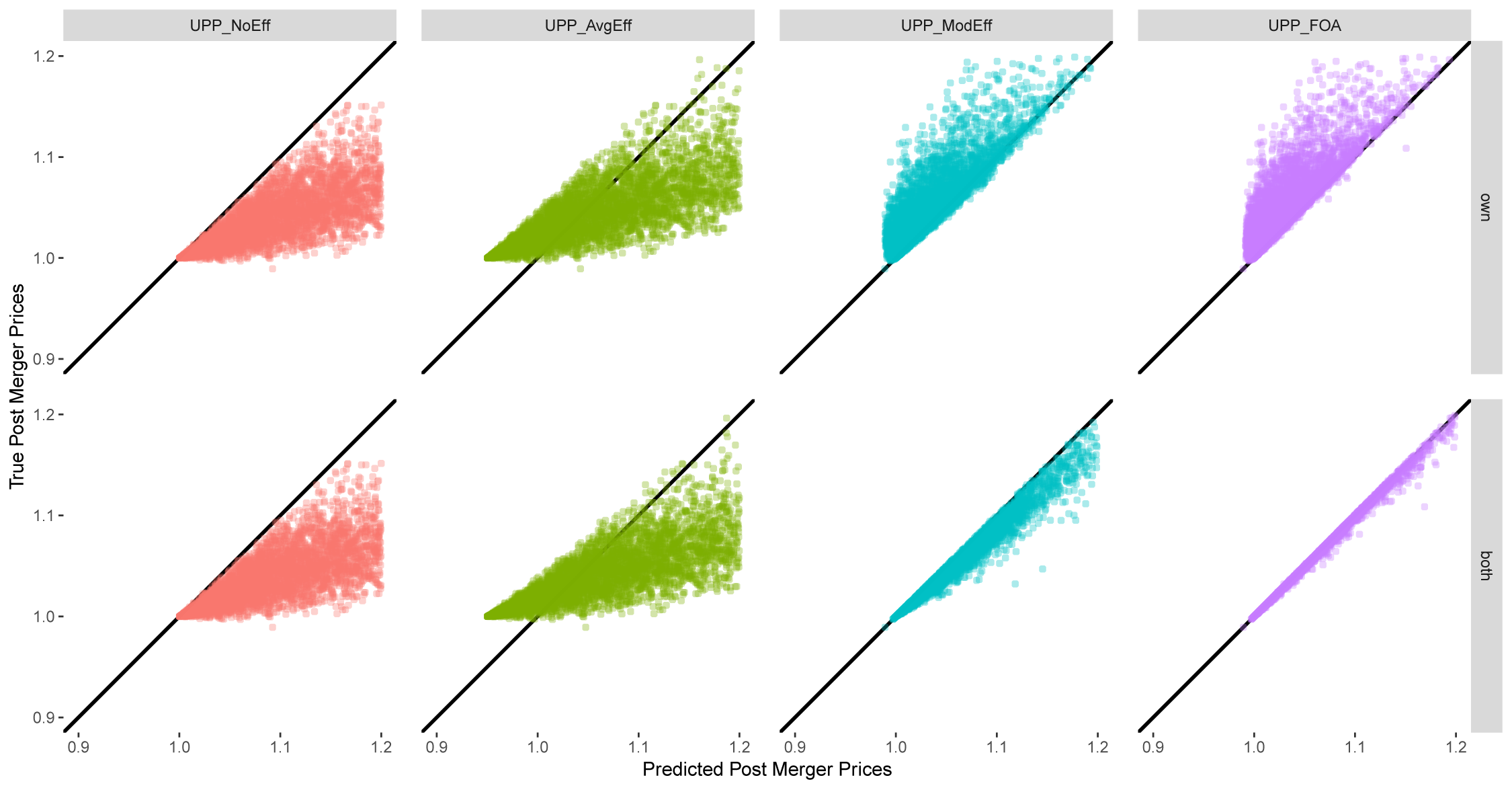

Supplement: S10 Fig — First row shows the distribution of the true post merger prices against the predicted post merger prices using different UPP calculations and own goods’ efficiencies included in the computation. Second row shows the same for both goods’ efficiencies. (TIF) [file pone.0227418.s010.tif]

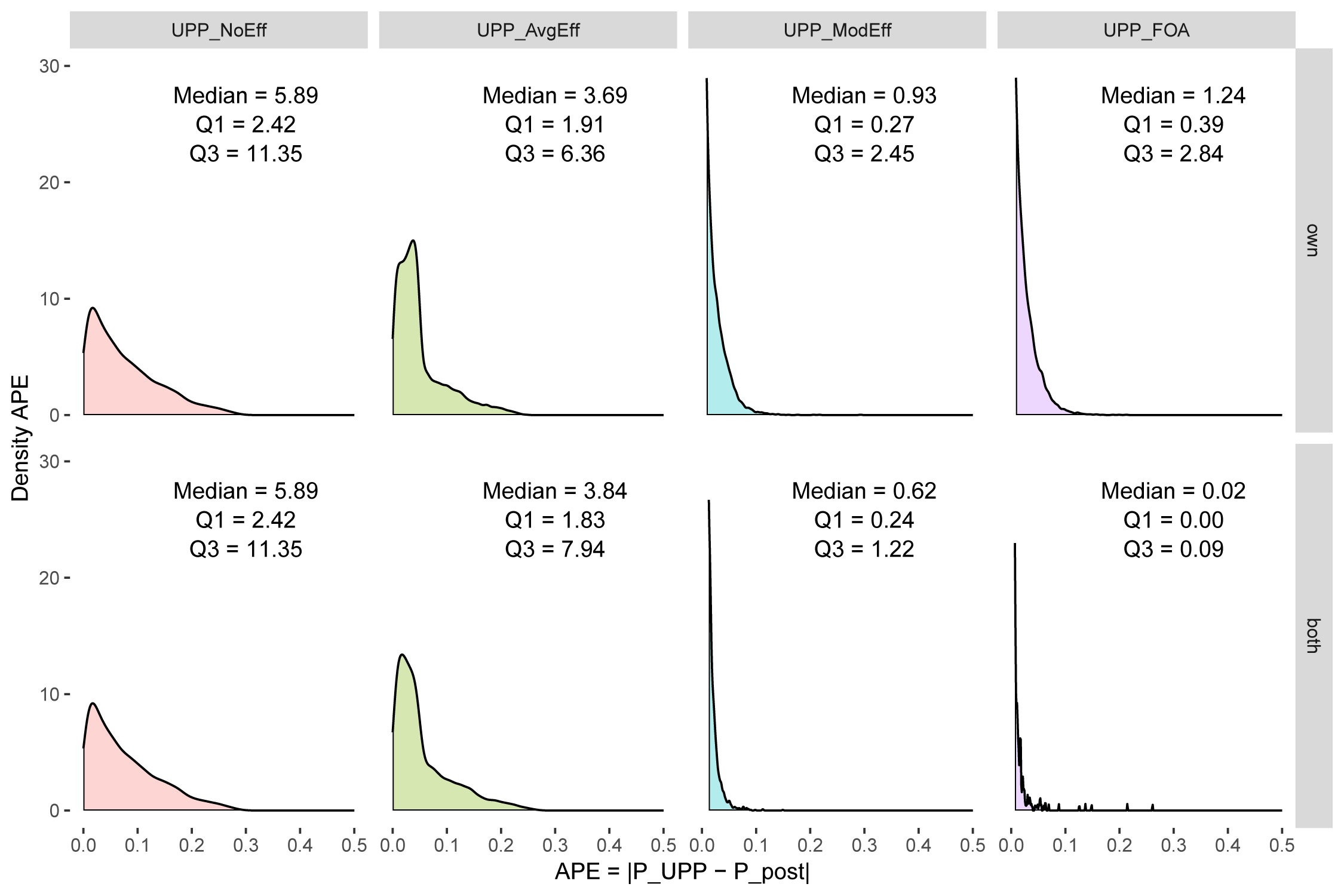

Supplement: S11 Fig — Portrays density kernels for absolute prediction errors, as well as the median absolute prediction error, first and third quartile for each specification. (TIF) [file pone.0227418.s011.tif]

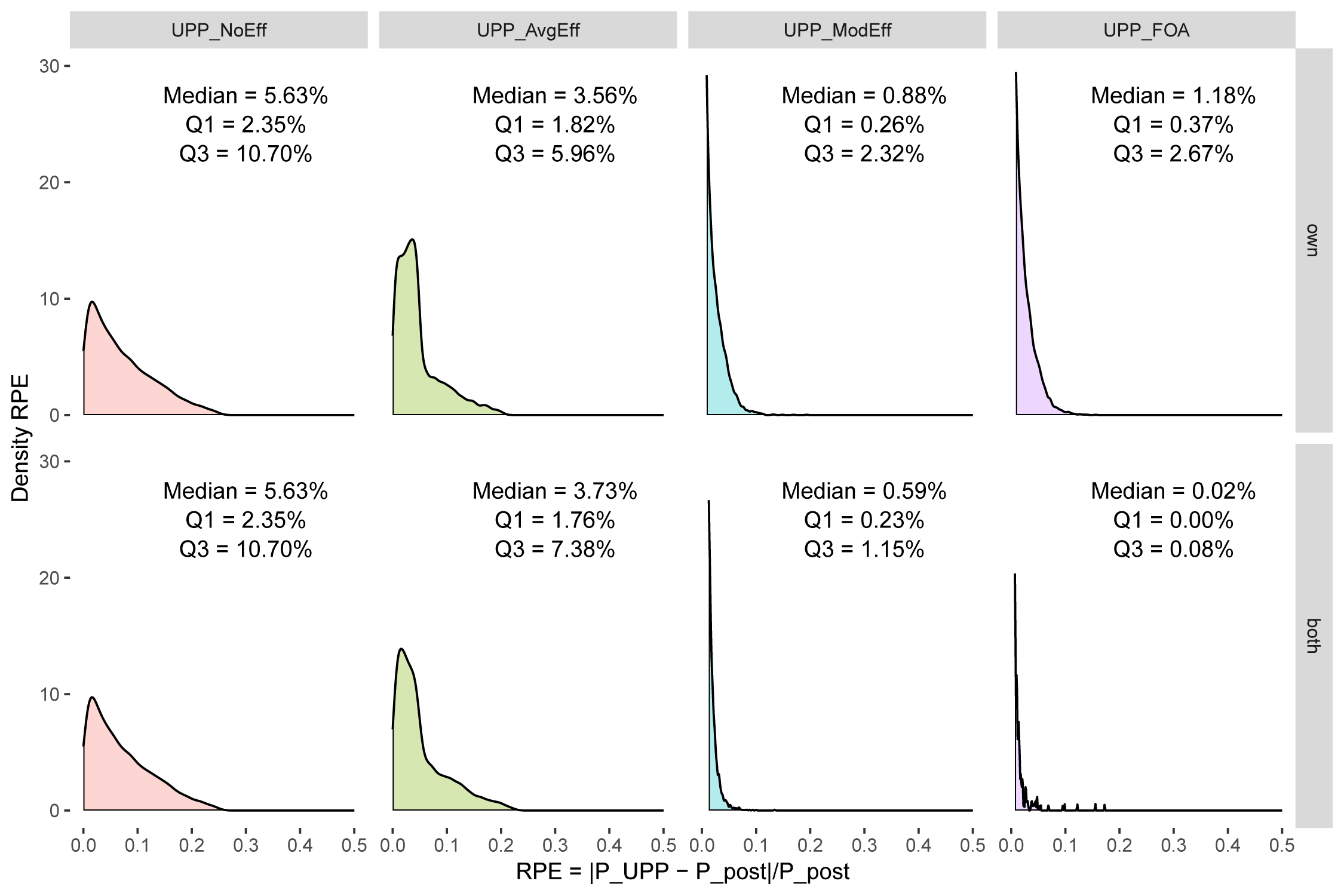

Supplement: S12 Fig — Portrays density kernels for relative prediction errors, as well as the median relative prediction error, first and third quartile for each specification. (TIF) [file pone.0227418.s012.tif]

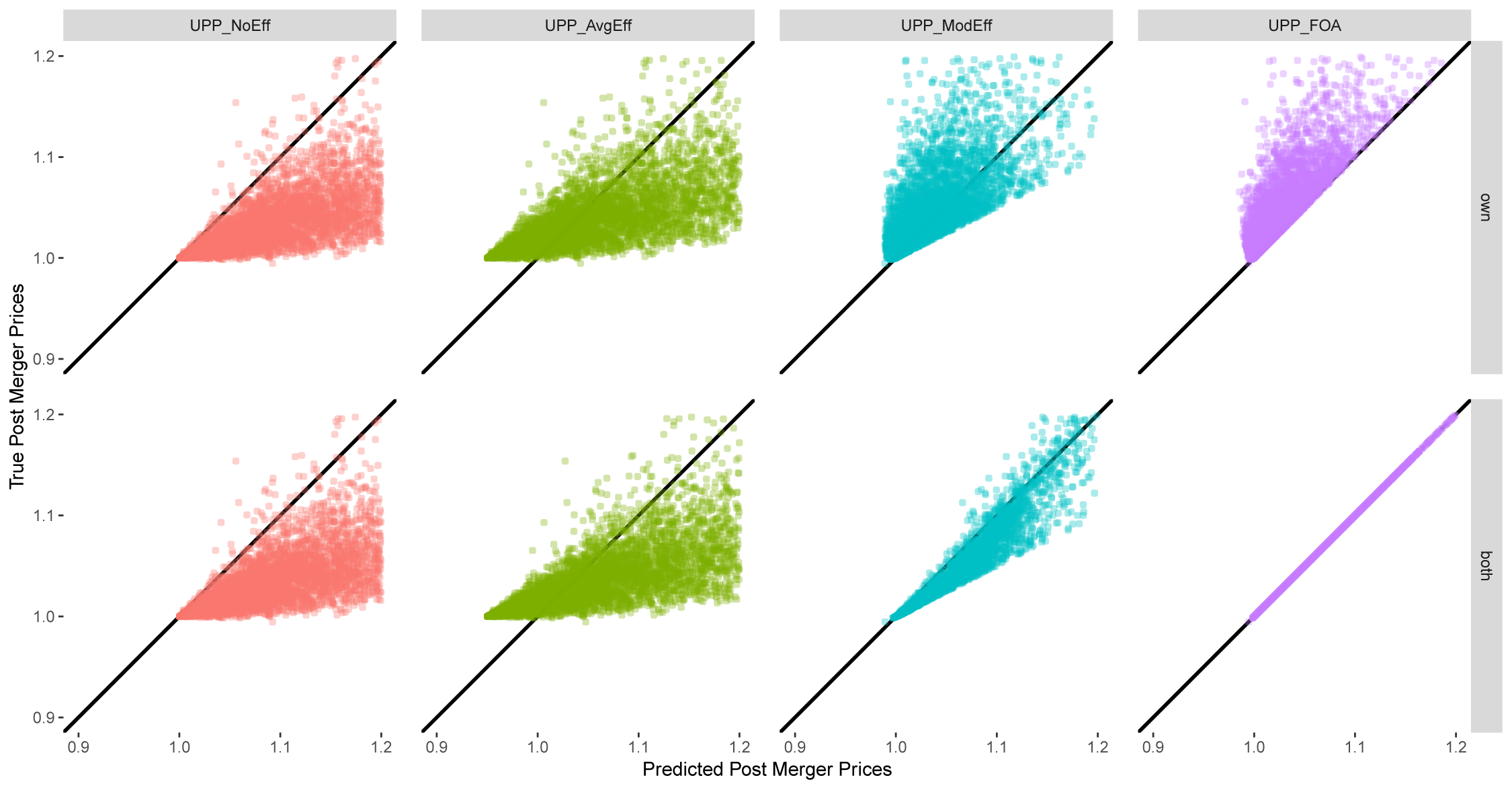

Supplement: S13 Fig — First row shows the distribution of the true post merger prices against the predicted post merger prices using different UPP calculations and own goods’ efficiencies included in the computation. Second row shows the same for both goods’ efficiencies. (TIF) [file pone.0227418.s013.tif]

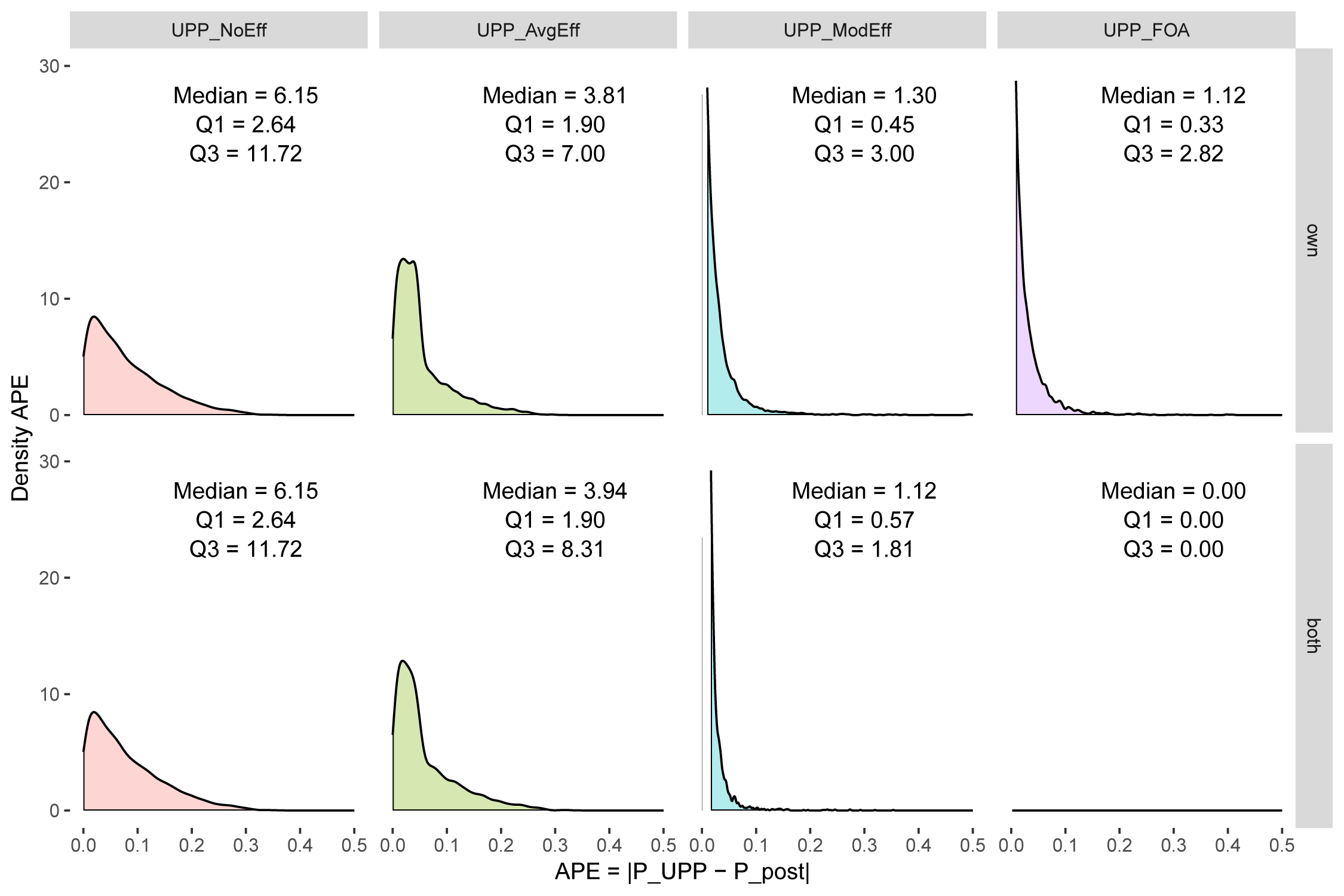

Supplement: S14 Fig — Portrays density kernels for absolute prediction errors, as well as the median absolute prediction error, first and third quartile for each specification. (TIF) [file pone.0227418.s014.tif]

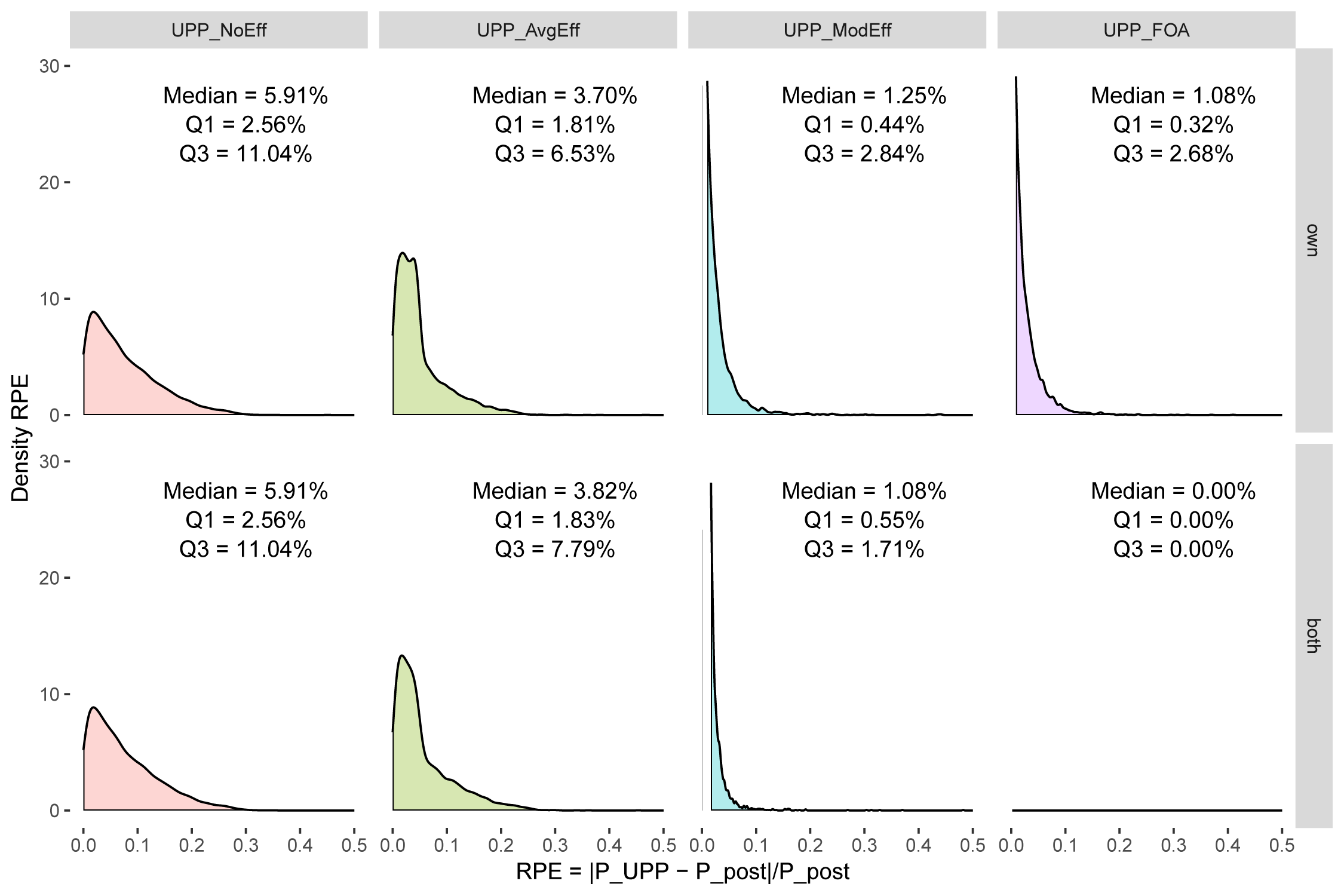

Supplement: S15 Fig — Portrays density kernels for relative prediction errors, as well as the median relative prediction error, first and third quartile for each specification. (TIF) [file pone.0227418.s015.tif]

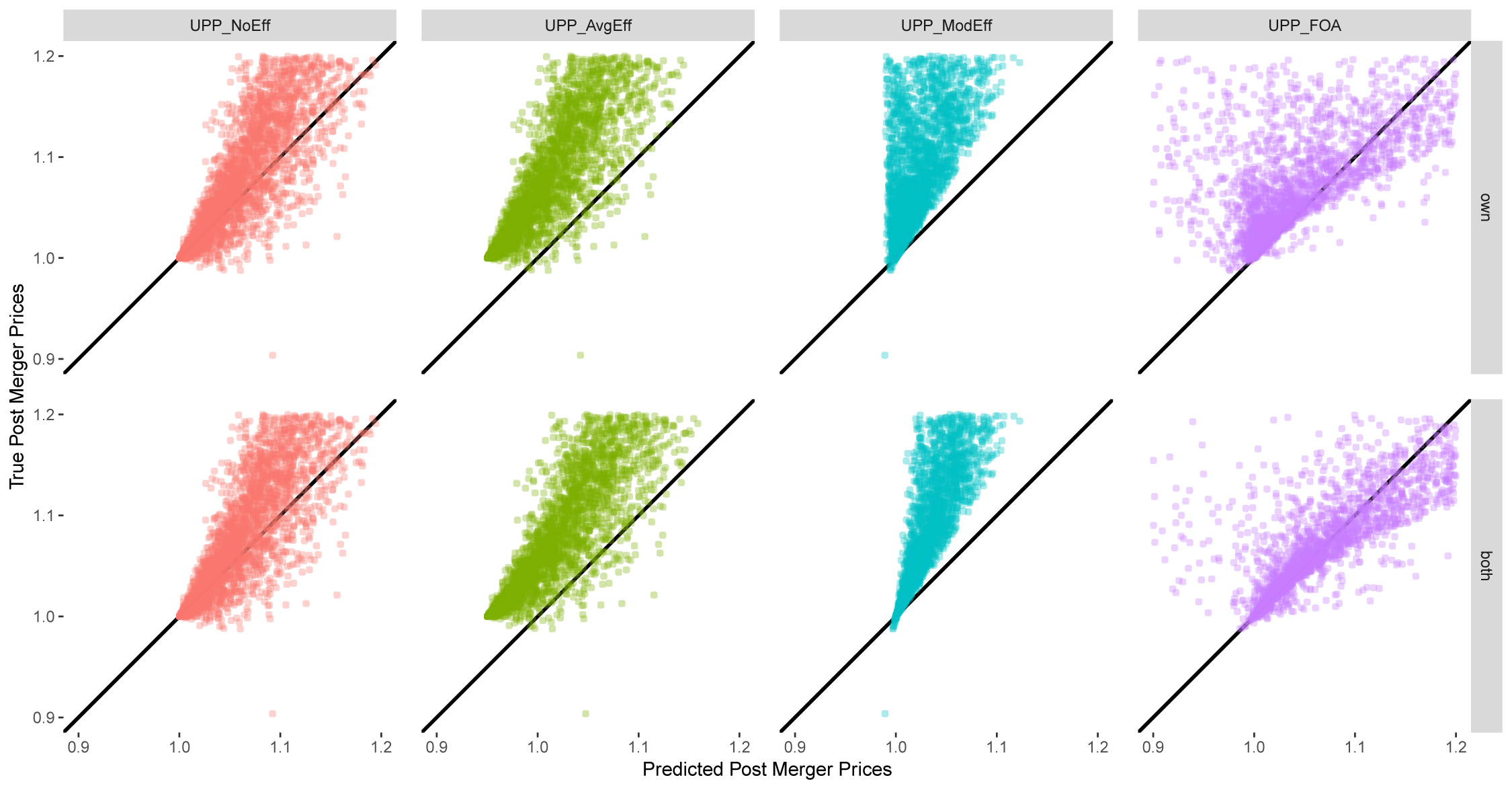

Supplement: S16 Fig — First row shows the distribution of the true post merger prices against the predicted post merger prices using different UPP calculations and own goods’ efficiencies included in the computation. Second row shows the same for both goods’ efficiencies. (TIF) [file pone.0227418.s016.tif]

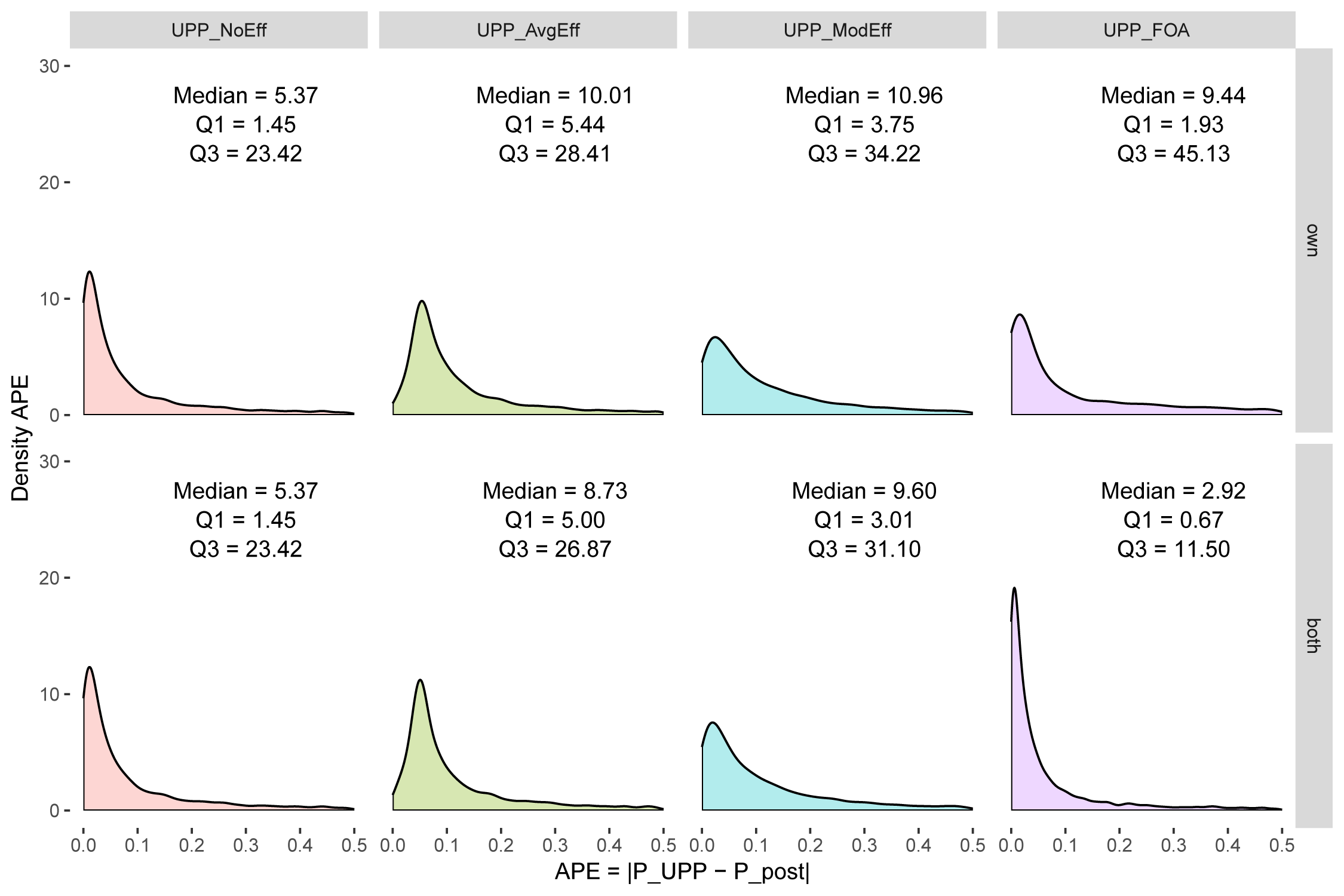

Supplement: S17 Fig — Portrays density kernels for absolute prediction errors, as well as the median absolute prediction error, first and third quartile for each specification. (TIF) [file pone.0227418.s017.tif]

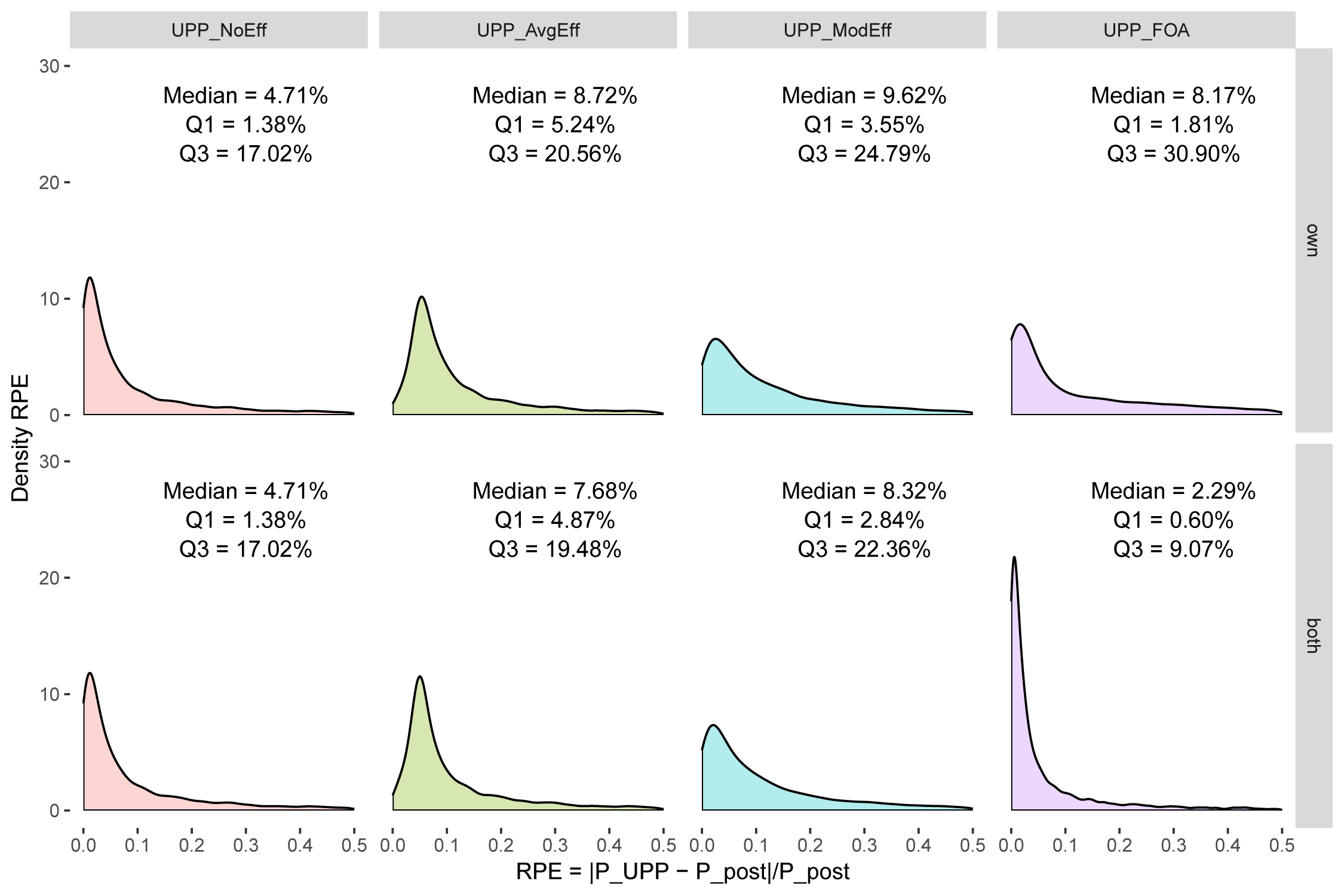

Supplement: S18 Fig — Portrays density kernels for relative prediction errors, as well as the median relative prediction error, first and third quartile for each specification. (TIF) [file pone.0227418.s018.tif]

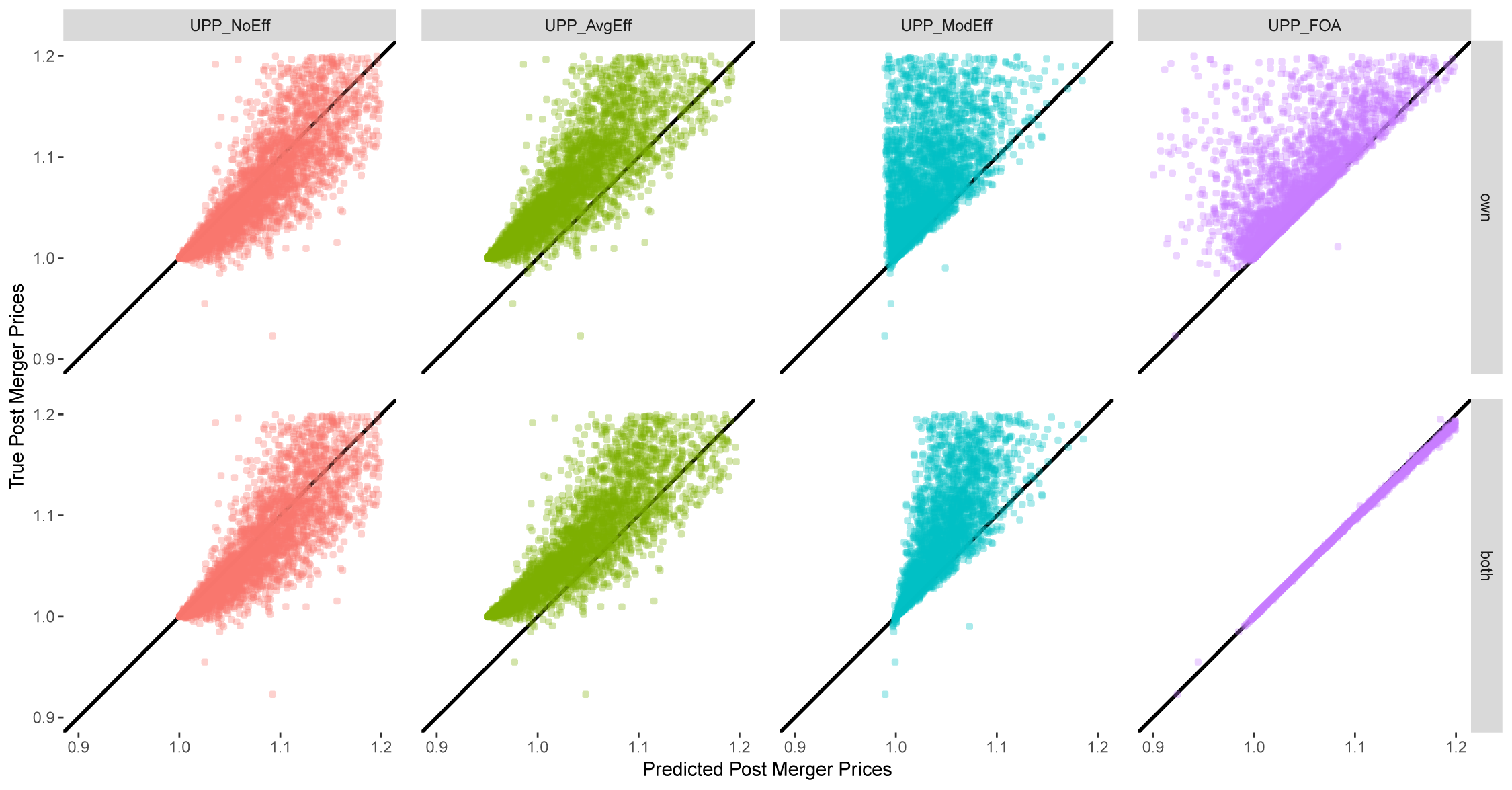

Supplement: S19 Fig — First row shows the distribution of the true post merger prices against the predicted post merger prices using different UPP calculations and own goods’ efficiencies included in the computation. Second row shows the same for both goods’ efficiencies. (TIF) [file pone.0227418.s019.tif]

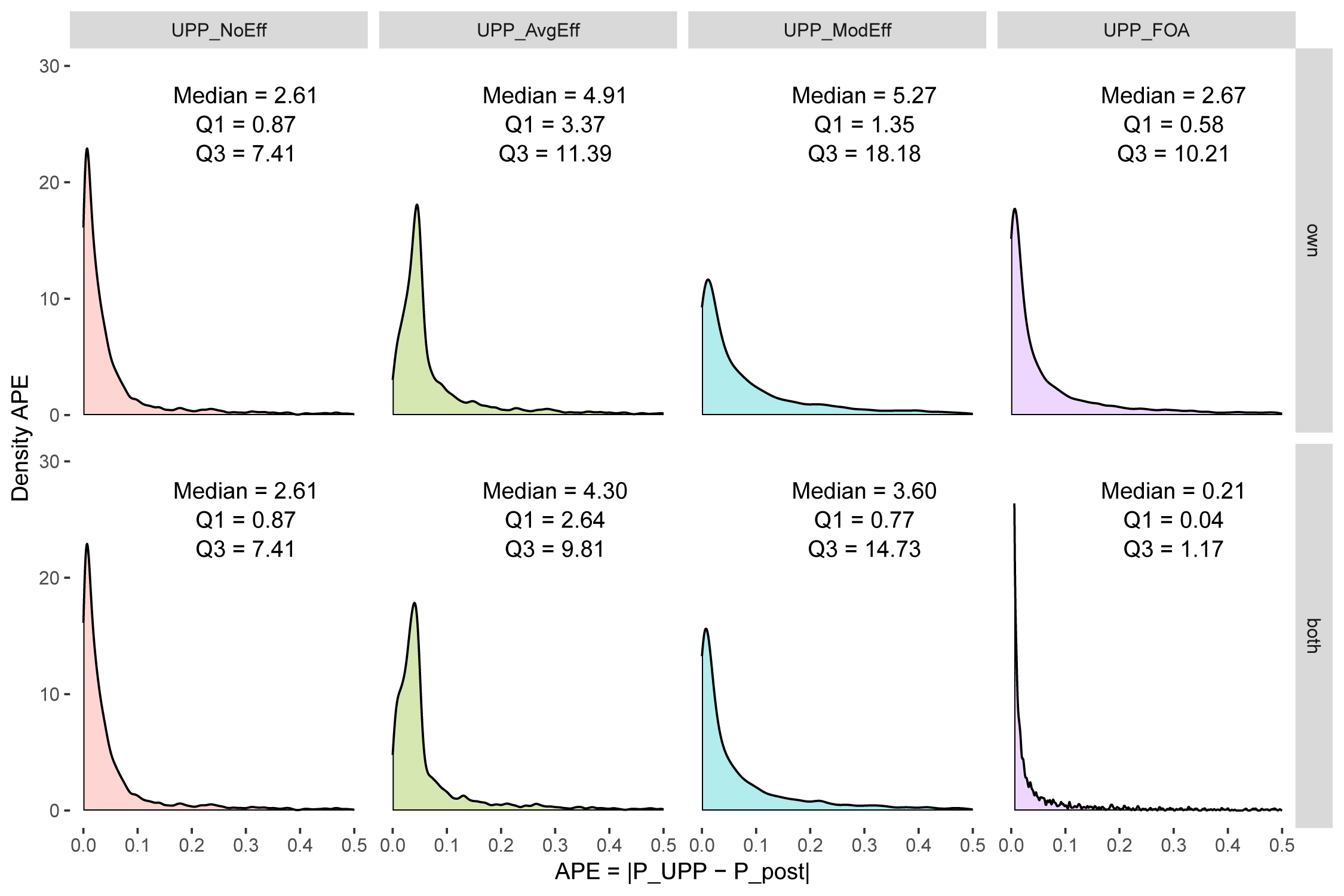

Supplement: S20 Fig — Portrays density kernels for absolute prediction errors, as well as the median absolute prediction error, first and third quartile for each specification. (TIF) [file pone.0227418.s020.tif]

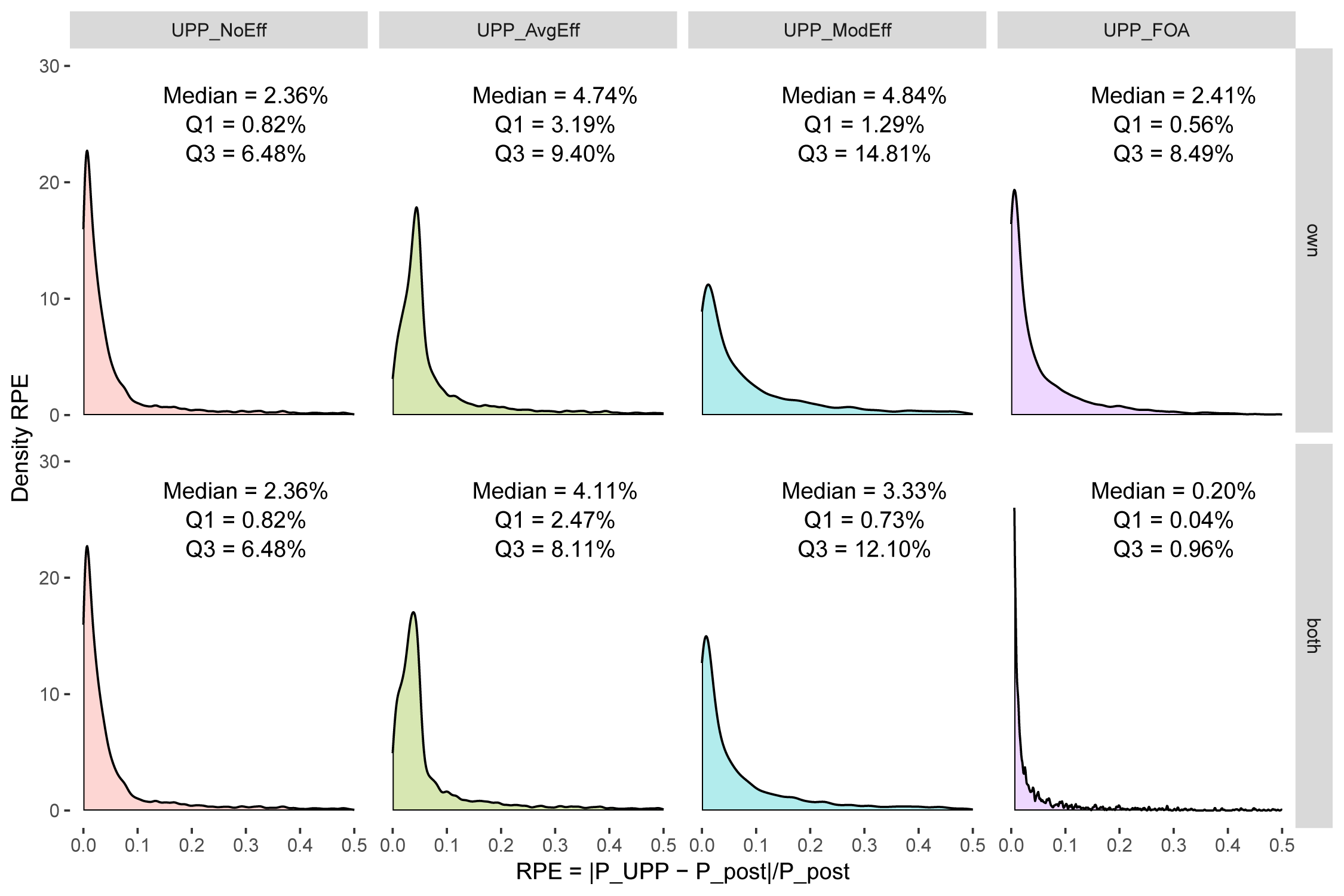

Supplement: S21 Fig — Portrays density kernels for relative prediction errors, as well as the median relative prediction error, first and third quartile for each specification. (TIF) [file pone.0227418.s021.tif]
