# Supplementary material for: Antitrust analysis with upward pricing pressure and cost efficiencies
Source: PLoS One. 2020 Jan 8;15(1):e0227418. doi: 10.1371/journal.pone.0227418 (PMC6949007; doi:10.1371/journal.pone.0227418)
Supplement: S1 Table — (PDF) [file pone.0227418.s023.pdf]

Generalized Leontief

Logit Demand	NoEff	AvgEff	ModEff	FOA
Type I error	0.436	0.123	0.026	0.007
Type II error	0.026	0.051	0.025	0.008
Total	0.462	0.175	0.050	0.015
Absolute Gain w.r.t. AvgEff			0.124	0.159
Relative Gain w.r.t. AvgEff (%)			71.11	91.22

Linear Demand	NoEff	AvgEff	ModEff	FOA
Type I error	0.433	0.120	0.026	0.000
Type II error	0.001	0.026	0.003	0.000
Total	0.434	0.147	0.029	0.000
Absolute Gain w.r.t. AvgEff			0.118	0.147
Relative Gain w.r.t. AvgEff (%)			80.56	100.00

Log-Linear Demand	NoEff	AvgEff	ModEff	FOA
Type I error	0.224	0.045	0.008	0.072
Type II error	0.044	0.182	0.193	0.038
Total	0.267	0.226	0.201	0.110
Absolute Gain w.r.t. AvgEff			0.025	0.116
Relative Gain w.r.t. AvgEff (%)			11.08	51.33

Almost Ideal Demand	NoEff	AvgEff	ModEff	FOA
Type I error	0.244	0.045	0.008	0.005
Type II error	0.024	0.145	0.138	0.010
Total	0.268	0.191	0.146	0.015
Absolute Gain w.r.t. AvgEff			0.045	0.176
Relative Gain w.r.t. AvgEff (%)			23.49	92.24

Quadratic

Logit Demand	NoEff	AvgEff	ModEff	FOA
Type I error	0.406	0.269	0.060	0.006
Type II error	0.000	0.002	0.000	0.000
Total	0.406	0.271	0.060	0.007
Absolute Gain w.r.t. AvgEff			0.211	0.264
Relative Gain w.r.t. AvgEff (%)			78.02	97.57

Linear Demand	NoEff	AvgEff	ModEff	FOA
Type I error	0.412	0.277	0.068	0.000
Type II error	0.003	0.005	0.004	0.000
Total	0.415	0.283	0.071	0.000
Absolute Gain w.r.t. AvgEff			0.211	0.283
Relative Gain w.r.t. AvgEff (%)			74.76	100.00

Log-Linear Demand	NoEff	AvgEff	ModEff	FOA
Type I error	0.048	0.033	0.021	0.031
Type II error	0.130	0.253	0.436	0.097
Total	0.178	0.286	0.457	0.128
Absolute Gain w.r.t. AvgEff			-0.171	0.157
Relative Gain w.r.t. AvgEff (%)			-59.84	55.11

Almost Ideal Demand	NoEff	AvgEff	ModEff	FOA
Type I error	0.096	0.042	0.019	0.009
Type II error	0.037	0.122	0.292	0.004
Total	0.133	0.165	0.311	0.013
Absolute Gain w.r.t. AvgEff			-0.146	0.152
Relative Gain w.r.t. AvgEff (%)			-88.60	92.31
