# Supplementary material for: Antitrust analysis with upward pricing pressure and cost efficiencies
Source: PLoS One. 2020 Jan 8;15(1):e0227418. doi: 10.1371/journal.pone.0227418 (PMC6949007; doi:10.1371/journal.pone.0227418)
Supplement: S2 Table — (PDF) [file pone.0227418.s024.pdf]

Generalized Leontief

Logit Demand	NoEff	AvgEff	ModEff	FOA
Precision Ratio	0.415	0.487	0.999	0.953
Recall Ratio	1.000	0.526	0.855	0.997
F1 score	0.587	0.506	0.921	0.975
Absolute Gain over AvgEff			0.395	0.469
Relative Gain over AvgEff (%)			82.15	92.76

Linear Demand	NoEff	AvgEff	ModEff	FOA
Precision Ratio	0.330	0.485	0.915	1.000
Recall Ratio	1.000	0.658	0.984	1.000
F1 score	0.497	0.559	0.949	1.000
Absolute Gain over AvgEff			0.390	0.441
Relative Gain over AvgEff (%)			69.77	78.99

Log-Linear Demand	NoEff	AvgEff	ModEff	FOA
Precision Ratio	0.395	0.419	0.734	0.646
Recall Ratio	1.000	0.476	0.661	0.653
F1 score	0.566	0.446	0.695	0.650
Absolute Gain over AvgEff			0.249	0.204
Relative Gain over AvgEff (%)			55.89	45.68

Almost Ideal Demand	NoEff	AvgEff	ModEff	FOA
Precision Ratio	0.400	0.447	0.790	0.872
Recall Ratio	1.000	0.501	0.702	0.725
F1 score	0.571	0.473	0.743	0.792
Absolute Gain over AvgEff			0.270	0.319
Relative Gain over AvgEff (%)			57.21	67.46

Quadratic

Logit Demand	NoEff	AvgEff	ModEff	FOA
Precision Ratio	0.996	0.999	1.000	1.000
Recall Ratio	1.000	0.796	1.000	1.000
F1 score	0.998	0.886	1.000	1.000
Absolute Gain over AvgEff			0.114	0.114
Relative Gain over AvgEff (%)			12.86	12.86

Linear Demand	NoEff	AvgEff	ModEff	FOA
Precision Ratio	0.993	0.998	0.998	1.000
Recall Ratio	1.000	0.798	1.000	1.000
F1 score	0.997	0.887	0.999	1.000
Absolute Gain over AvgEff			0.112	0.113
Relative Gain over AvgEff (%)			12.63	12.75

Log-Linear Demand	NoEff	AvgEff	ModEff	FOA
Precision Ratio	0.962	0.934	0.966	0.997
Recall Ratio	1.000	0.771	1.000	0.836
F1 score	0.980	0.845	0.983	0.909
Absolute Gain over AvgEff			0.138	0.065
Relative Gain over AvgEff (%)			16.32	7.67

Almost Ideal Demand	NoEff	AvgEff	ModEff	FOA
Precision Ratio	0.964	0.947	0.968	0.998
Recall Ratio	1.000	0.780	0.999	0.952
F1 score	0.982	0.855	0.984	0.974
Absolute Gain over AvgEff			0.128	0.119
Relative Gain over AvgEff (%)			15.01	13.93
