# Supplementary material for: Antitrust analysis with upward pricing pressure and cost efficiencies
Source: PLoS One. 2020 Jan 8;15(1):e0227418. doi: 10.1371/journal.pone.0227418 (PMC6949007; doi:10.1371/journal.pone.0227418)
Supplement: S3 Table — (PDF) [file pone.0227418.s025.pdf]

Generalized Leontief

Logit Demand	NoEff	AvgEff	ModEff	FOA
Precision Ratio	0.180	0.362	0.789	0.936
Recall Ratio	0.787	0.576	0.795	0.933
F1 score	0.293	0.445	0.792	0.935
Absolute Gain over AvgEff			0.216	0.490
Relative Gain over AvgEff (%)			78.18	110.20

Linear Demand	NoEff	AvgEff	ModEff	FOA
Precision Ratio	0.186	0.379	0.791	1.000
Recall Ratio	0.992	0.735	0.971	1.000
F1 score	0.313	0.500	0.872	1.000
Absolute Gain over AvgEff			0.372	0.500
Relative Gain over AvgEff (%)			74.32	100.00

Log-Linear Demand	NoEff	AvgEff	ModEff	FOA
Precision Ratio	0.379	0.350	0.515	0.609
Recall Ratio	0.682	0.229	0.213	0.628
F1 score	0.487	0.277	0.301	0.619
Absolute Gain over AvgEff			0.025	0.342
Relative Gain over AvgEff (%)			8.97	123.67

Almost Ideal Demand	NoEff	AvgEff	ModEff	FOA
Precision Ratio	0.365	0.382	0.678	0.847
Recall Ratio	0.750	0.286	0.321	0.700
F1 score	0.491	0.327	0.436	0.767
Absolute Gain over AvgEff			0.109	0.440
Relative Gain over AvgEff (%)			33.24	134.54

Quadratic

Logit Demand	NoEff	AvgEff	ModEff	FOA
Precision Ratio	0.238	0.317	0.680	0.952
Recall Ratio	1.000	0.988	1.000	0.998
F1 score	0.384	0.480	0.809	0.975
Absolute Gain over AvgEff			0.330	0.495
Relative Gain over AvgEff (%)			68.74	103.16

Linear Demand	NoEff	AvgEff	ModEff	FOA
Precision Ratio	0.225	0.297	0.637	1.000
Recall Ratio	0.979	0.956	0.968	1.000
F1 score	0.366	0.453	0.769	1.000
Absolute Gain over AvgEff			0.316	0.547
Relative Gain over AvgEff (%)			69.77	120.90

Log-Linear Demand	NoEff	AvgEff	ModEff	FOA
Precision Ratio	0.825	0.771	0.613	0.934
Recall Ratio	0.755	0.523	0.196	0.760
F1 score	0.788	0.623	0.297	0.838
Absolute Gain over AvgEff			-0.326	0.215
Relative Gain over AvgEff (%)			-52.32	34.49

Almost Ideal Demand	NoEff	AvgEff	ModEff	FOA
Precision Ratio	0.767	0.804	0.764	0.964
Recall Ratio	0.912	0.709	0.318	0.947
F1 score	0.833	0.753	0.449	0.956
Absolute Gain over AvgEff			-0.304	0.202
Relative Gain over AvgEff (%)			-40.41	26.83
