# Supplementary material for: Antitrust analysis with upward pricing pressure and cost efficiencies
Source: PLoS One. 2020 Jan 8;15(1):e0227418. doi: 10.1371/journal.pone.0227418 (PMC6949007; doi:10.1371/journal.pone.0227418)
Supplement: S4 Table — (PDF) [file pone.0227418.s026.pdf]

Curvature - Total Errors

Logit Demand	NoEff	AvgEff	ModEff	FOA
Type I error	0.454	0.199	0.001	0.011
Type II error	0.000	0.290	0.036	0.002
Total	0.454	0.489	0.037	0.013
Absolute Gain over AvgEff			0.451	0.475
Relative Gain over AvgEff (%)			92.37	97.26

Linear Demand	NoEff	AvgEff	ModEff	FOA
Type I error	0.521	0.195	0.041	0.000
Type II error	0.000	0.218	0.009	0.000
Total	0.521	0.413	0.050	0.000
Absolute Gain over AvgEff			0.362	0.413
Relative Gain over AvgEff (%)			87.82	100.00

Log-Linear Demand	NoEff	AvgEff	ModEff	FOA
Type I error	0.418	0.128	0.057	0.081
Type II error	0.000	0.249	0.054	0.059
Total	0.418	0.378	0.111	0.141
Absolute Gain over AvgEff			0.266	0.237
Relative Gain over AvgEff (%)			70.55	62.70

Almost Ideal Demand	NoEff	AvgEff	ModEff	FOA
Type I error	0.413	0.122	0.052	0.008
Type II error	0.000	0.245	0.037	0.011
Total	0.413	0.367	0.089	0.019
Absolute Gain over AvgEff			0.278	0.349
Relative Gain over AvgEff (%)			75.69	94.89

Curvature - F1 score

Logit Demand	NoEff	AvgEff	ModEff	FOA
Precision Ratio	0.546	0.563	0.998	0.979
Recall Ratio	1.000	0.469	0.933	0.996
F1 score	0.707	0.512	0.964	0.987
Absolute Gain over AvgEff			0.452	0.476
Relative Gain over AvgEff (%)			88.39	92.92

Linear Demand	NoEff	AvgEff	ModEff	FOA
Precision Ratio	0.479	0.573	0.920	1.000
Recall Ratio	1.000	0.545	0.981	1.000
F1 score	0.648	0.558	0.949	1.000
Absolute Gain over AvgEff			0.391	0.442
Relative Gain over AvgEff (%)			69.98	79.07

Log-Linear Demand	NoEff	AvgEff	ModEff	FOA
Precision Ratio	0.582	0.561	0.832	0.799
Recall Ratio	1.000	0.439	0.730	0.716
F1 score	0.736	0.492	0.777	0.755
Absolute Gain over AvgEff			0.285	0.263
Relative Gain over AvgEff (%)			57.92	53.42

Almost Ideal Demand	NoEff	AvgEff	ModEff	FOA
Precision Ratio	0.587	0.588	0.863	0.929
Recall Ratio	1.000	0.456	0.751	0.804
F1 score	0.740	0.514	0.803	0.862
Absolute Gain over AvgEff			0.290	0.349
Relative Gain over AvgEff (%)			56.43	67.90
