# Supplementary material for: Antitrust analysis with upward pricing pressure and cost efficiencies
Source: PLoS One. 2020 Jan 8;15(1):e0227418. doi: 10.1371/journal.pone.0227418 (PMC6949007; doi:10.1371/journal.pone.0227418)
Supplement: S5 Table — (PDF) [file pone.0227418.s027.pdf]

Curvature - Total Errors

Logit Demand	NoEff	AvgEff	ModEff	FOA
Type I error	0.467	0.146	0.027	0.004
Type II error	0.015	0.027	0.015	0.006
Total	0.482	0.174	0.042	0.010
Absolute Gain over AvgEff			0.132	0.163
Relative Gain over AvgEff (%)			75.95	93.96

Linear Demand	NoEff	AvgEff	ModEff	FOA
Type I error	0.457	0.141	0.020	0.000
Type II error	0.002	0.020	0.006	0.000
Total	0.459	0.161	0.026	0.000
Absolute Gain over AvgEff			0.135	0.161
Relative Gain over AvgEff (%)			83.89	100.00

Log-Linear Demand	NoEff	AvgEff	ModEff	FOA
Type I error	0.205	0.052	0.011	0.076
Type II error	0.059	0.228	0.277	0.045
Total	0.265	0.280	0.288	0.121
Absolute Gain over AvgEff			-0.008	0.159
Relative Gain over AvgEff (%)			-2.91	56.81

Almost Ideal Demand	NoEff	AvgEff	ModEff	FOA
Type I error	0.248	0.052	0.009	0.005
Type II error	0.017	0.139	0.171	0.011
Total	0.265	0.190	0.180	0.017
Absolute Gain over AvgEff			0.010	0.174
Relative Gain over AvgEff (%)			5.38	91.25

Curvature - F1 score

Logit Demand	NoEff	AvgEff	ModEff	FOA
Precision Ratio	0.122	0.263	0.705	0.939
Recall Ratio	0.814	0.655	0.816	0.919
F1 score	0.212	0.375	0.757	0.929
Absolute Gain over AvgEff			0.381	0.553
Relative Gain over AvgEff (%)			101.57	147.41

Linear Demand	NoEff	AvgEff	ModEff	FOA
Precision Ratio	0.142	0.289	0.781	1.000
Recall Ratio	0.970	0.738	0.925	1.000
F1 score	0.247	0.416	0.847	1.000
Absolute Gain over AvgEff			0.431	0.584
Relative Gain over AvgEff (%)			103.75	140.54

Log-Linear Demand	NoEff	AvgEff	ModEff	FOA
Precision Ratio	0.475	0.444	0.577	0.699
Recall Ratio	0.712	0.248	0.150	0.693
F1 score	0.570	0.318	0.238	0.696
Absolute Gain over AvgEff			-0.080	0.378
Relative Gain over AvgEff (%)			-25.21	118.77

Almost Ideal Demand	NoEff	AvgEff	ModEff	FOA
Precision Ratio	0.406	0.472	0.758	0.844
Recall Ratio	0.821	0.356	0.266	0.772
F1 score	0.543	0.406	0.394	0.806
Absolute Gain over AvgEff			-0.013	0.400
Relative Gain over AvgEff (%)			-3.10	98.55
